# HOXC10 Protects from Skin Aging by Targeting the FZD6/Wnt/β-Catenin Signaling Pathway

**DOI:** 10.34133/research.0976

**Published:** 2025-11-19

**Authors:** Yun Zhong, Yi Guo, Rui Mao, Lei Zhou, Fan Wang, Xin Meng, Xin Xiao, Haonan Yuan, Yifan Zhang, Zhili Deng, Wei Shi, Qian Wang, Hongfu Xie, Yiya Zhang, Ji Li

**Affiliations:** ^1^ Department of Dermatology, Xiangya Hospital, Central South University, Changsha, P.R. China.; ^2^ Hunan Key Laboratory of Aging Biology, Xiangya Hospital, Central South University, Changsha, P.R. China.; ^3^ Department of Dermatology, Nanfang Hospital, Southern Medical University, Guangzhou, P.R. China.; ^4^ Department of Dermatology, The Third Affiliated Hospital, Sun Yat-sen University, Guangzhou, P.R. China.; ^5^ National Clinical Research Center for Geriatric Disorders, Xiangya Hospital, Central South University, Changsha 410008, Hunan, P.R. China.; ^6^ Hunan Binsis Biotechnology Co., Ltd., Changsha, P.R. China.; ^7^ The First Hospital of Changsha, Changsha 410005, P.R. China.; ^8^ The Affiliated Changsha Hospital of Xiangya School of Medicine, Central South University, Changsha 410008, P.R. China.

## Abstract

Aging, caused by a variety of exogenous stimuli and internal factors, leads to a gradual and irreversible decline in the function of the organism. The aging process involves complex gene regulation, in which transcription factors may play a key role as core regulatory elements. In this study, the single-cell transcriptome of skin revealed homeobox C10 (HOXC10) as a core element in the transcription factor regulatory network associated with skin aging. In vivo and vitro, the expression of HOXC10 was down-regulated in senescent fibroblasts and aging tissues, and overexpressed HOXC10 delayed cell senescence and skin aging. Mechanistically, HOXC10 targeted the promoter region of frizzled 6 (FZD6) to reduce its expression and therefore activated the Wnt/β-catenin signaling pathway to delay aging. Finally, by using the Connectivity Map approach, we explored simvastatin as a functional mimic of HOXC10 and demonstrated its antiaging ability in vitro and vivo. In conclusion, our results establish the role of HOXC10/FZD6/Wnt signals in skin aging and identify simvastatin as a potential therapeutic strategy to delay aging.

## Introduction

Aging is characterized by a gradual decline in the function of tissues and organs, a process driven by a combination of internal and external factors. This deterioration considerably contributes to the onset of age-related diseases, including cardiovascular and neurodegenerative disorders [1]. Among the various manifestations of aging, skin aging is particularly prominent and easily noticeable. Serving as a protective barrier against environmental stressors, the skin not only is impacted by intrinsic and chronological aging but is also highly vulnerable to environmental factors that accelerate aging. Additionally, studies suggest that skin aging may play a pivotal role in the broader aging process and contribute to the functional impairments commonly associated with aging [2].

The accumulation of senescent cells is widely considered a primary driver of organ aging. Various factors can trigger cellular senescence, including telomere shortening, exposure to radiation, chemical agents, activation of oncogenes, and mitochondrial dysfunction [3]. The induction and persistence of cellular senescence are governed by intricate gene regulation mechanisms and multiple signaling pathways, such as those involving mTOR, NF-κB, and Wnt [4–8]. A hallmark of senescence is cell cycle arrest, which is primarily mediated through the up-regulation of the p53/p21 and p16/pRB pathways [9]. Senescent cells also release a range of bioactive factors, collectively known as the senescence-associated secretory phenotype (SASP) [10,11], which can instigate local inflammation and promote the senescence of neighboring cells [10,12]. Fibroblasts, which constitute the bulk of dermal connective tissue, are a major source of senescent cells in aging skin [13–16]. These fibroblasts are crucial for synthesizing the extracellular matrix and communicating with adjacent cells, thereby contributing considerably to the maintenance of the skin’s structural integrity and function.

As the core elements of gene regulation, transcription factors play important regulatory roles during aging [17–19]. By modulating the activity of these transcription factors, it is possible to control the expression of genes associated with aging, offering potential strategies for slowing down the aging process. Homeobox C10 (HOXC10), a member of the homeobox (HOX) family of transcription factors, is primarily involved in developmental regulation [20]. Aberrant expression of HOXC10 has been associated with increased cell proliferation and migration in several cancers, including gastric, ovarian, and cervical cancers and melanoma [21–23]. Moreover, HOXC10 regulates the cell cycle by affecting mitotic progression [24]. Despite its potential to influence cell proliferation and the cell cycle, the impact of HOXC10 on cell senescence remains poorly understood.

In this study, we explored the protective function of HOXC10 against skin aging and demonstrated that HOXC10 activates the canonical Wnt/β-catenin signaling pathway by suppressing frizzled 6 (FZD6) transcription, thereby delaying the cell senescence and skin aging processes. Additionally, we identified simvastatin (SIM) as a novel compound that mimics the effects of HOXC10, delaying skin aging. In summary, our findings emphasize the critical role of the HOXC10/FZD6 axis in skin aging and propose SIM as a promising therapeutic approach for combating age-related skin deterioration.

## Results

### HOXC10 expression is reduced in both aged skin and senescent HDFs

**Fig. 1.**
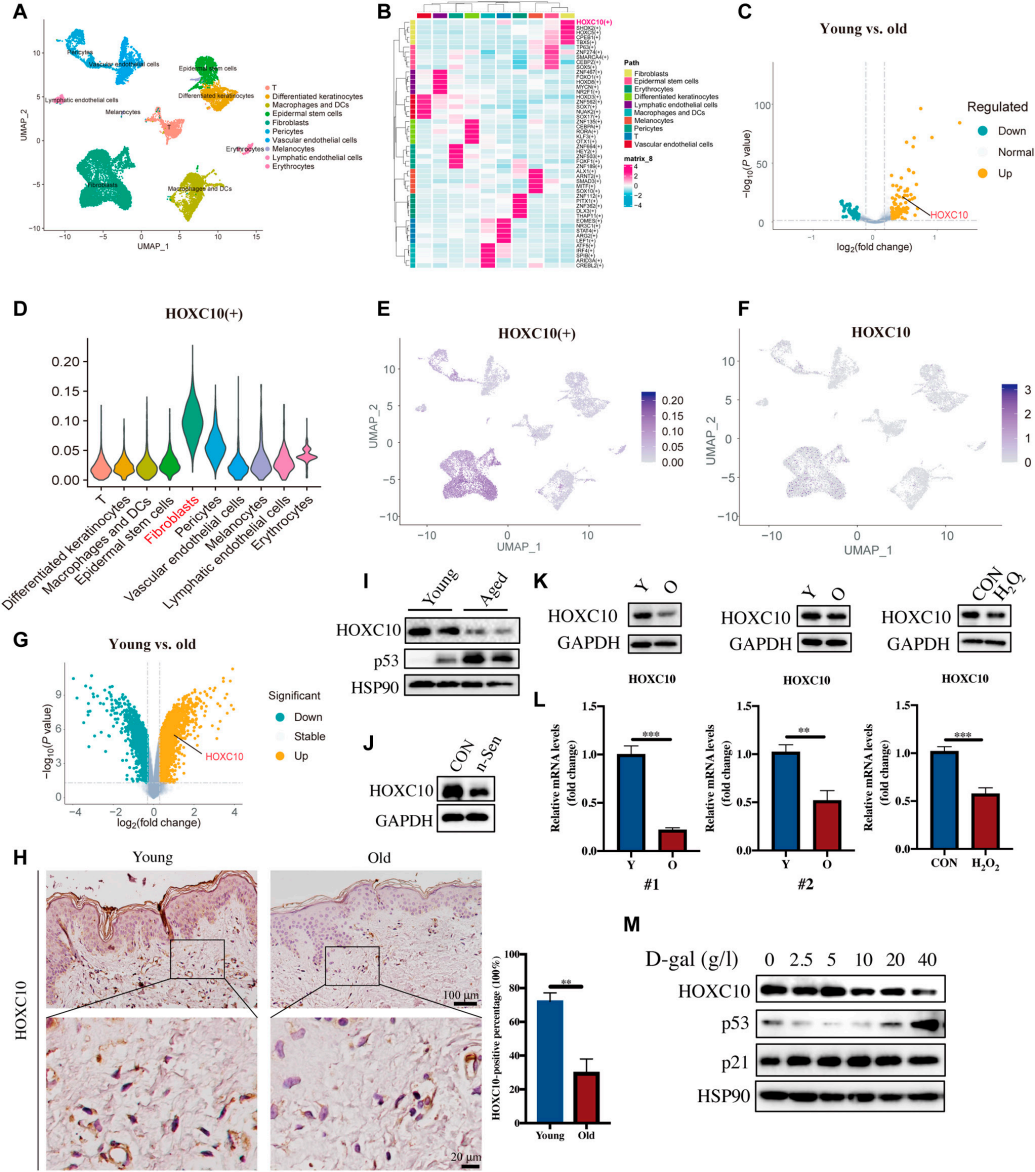
Homeobox C10 (HOXC10) plays an important role in senescent cells and aging skin tissue. (A) Two-dimensional uniform manifold approximation and projection (UMAP) visualization of the single-cell transcriptome in skin aging: Each point symbolizes an individual cell, with colors indicating the species of each cell. (B) Multifunctional heatmaps derived from transcription factor regulatory network analysis: Varied color blocks correspond to distinct cells as annotated in the graph. (C) Comparative analysis of young and aged samples in fibroblast populations from single-cell transcriptomic subsets: Each point represents a gene, with yellow signifying up-regulation in the young group and green indicating up-regulation in the aged group (logFC = 0.53). (D) Vlnplot illustrating HOXC10 regulatory activity from transcription factor network analysis: Diverse color blocks denote different cell populations; (+) represents the transcriptional activity of HOXC10. (E) Featureplot of HOXC10 regulatory activity based on transcription factor network analysis: Each point indicates a cell, with darker hues correlating with higher activity levels; (+) represents the transcriptional activity of HOXC10. (F) Featureplot of HOXC10 messenger RNA (mRNA) expression: Individual points represent cells, where darker colors reflect higher expression levels. (G) Differential gene expression analysis in the bulk transcriptome of human skin tissues across age groups: Each point is a gene, with yellow illustrating up-regulation in the young cohort and green depicting up-regulation in the aged cohort (logFC = 1.1). (H) Immunohistochemistry (IHC) staining of HOXC10 in the human skin of the young (*n* = 8, median age 23 years, range 16 to 28) and old groups (*n* = 6, median age 62 years, range 53 to 74). For each donor, 3 to 5 nonoverlapping microscopic fields were analyzed; at least 100 cells per donor were quantified. Scale bars: 100 μm. (I) Western blotting showed the expression levels of HOXC10 in young (6 to 8 weeks) and aged mouse skin (16 to 18 months). All results are representative results from at least 3 repeated independent experiments. (J to M) Human dermal fibroblasts (HDFs) were used to construct nutlin-3a senescence, passage senescence (#1, #2, 2 different individuals; Y, young passage; O, old passage), H_2_O_2_-induced senescence, and D-galactose (D-gal)-induced senescence. After verifying the successful model construction, the expression of HOXC10 in senescent HDFs was analyzed by RT-qPCR and western blotting. The data are shown as mean ± standard error of the mean (SEM); **P ≤ 0.01; ***P ≤ 0.001. DCs, dendritic cells; GAPDH, glyceraldehyde-3-phosphate dehydrogenase; CON, control.

To explore the key regulators in skin aging, we analyzed the transcription factor regulatory network in single-cell datasets of human young/aging skin (GSE130973). As shown in Fig. 1A to E, the transcription factor activity of HOXC10 is specifically observed in fibroblasts and is significantly down-regulated in the fibroblasts of aged skin (Fig. 1A to F and Figs. S1A to C and S2). Down-regulated expression of HOXC10 was also observed in the fibroblasts of aged skin using single-cell transcriptome (GSE130973) and bulk transcriptome data (GSE64553) (Fig. 1C and G).

Subsequently, we validated HOXC10 expression in human and mouse aging skin. Compared to that in the young group, HOXC10 expression was significantly reduced in the skin of older individuals (Fig. 1H). Likewise, similar results were found in mouse skin tissue (Fig. 1I). Furthermore, we constructed multiple cellular senescence models in vitro, including replicative senescence, H_2_O_2_, nutlin-3a (n-Sen) [25], and D-galactose (D-gal)-induced senescence. All senescence models were verified with aging-related markers, such as increased levels of p53, p21, and p16 proteins, or increased staining of senescence-related galactosidase, to ensure the success of aging model construction (Fig. S1D to G and I). Our findings demonstrated a significant reduction in HOXC10 expression across all senescence models (Fig. 1J to M and Fig. S1H). Together, these findings suggest that HOXC10 expression is down-regulated in both aging skin and senescent human dermal fibroblasts (HDFs), implying its potential involvement in the aging process.

### Down-regulated HOXC10 promotes cellular senescence and accelerates skin aging

**Fig. 2.**
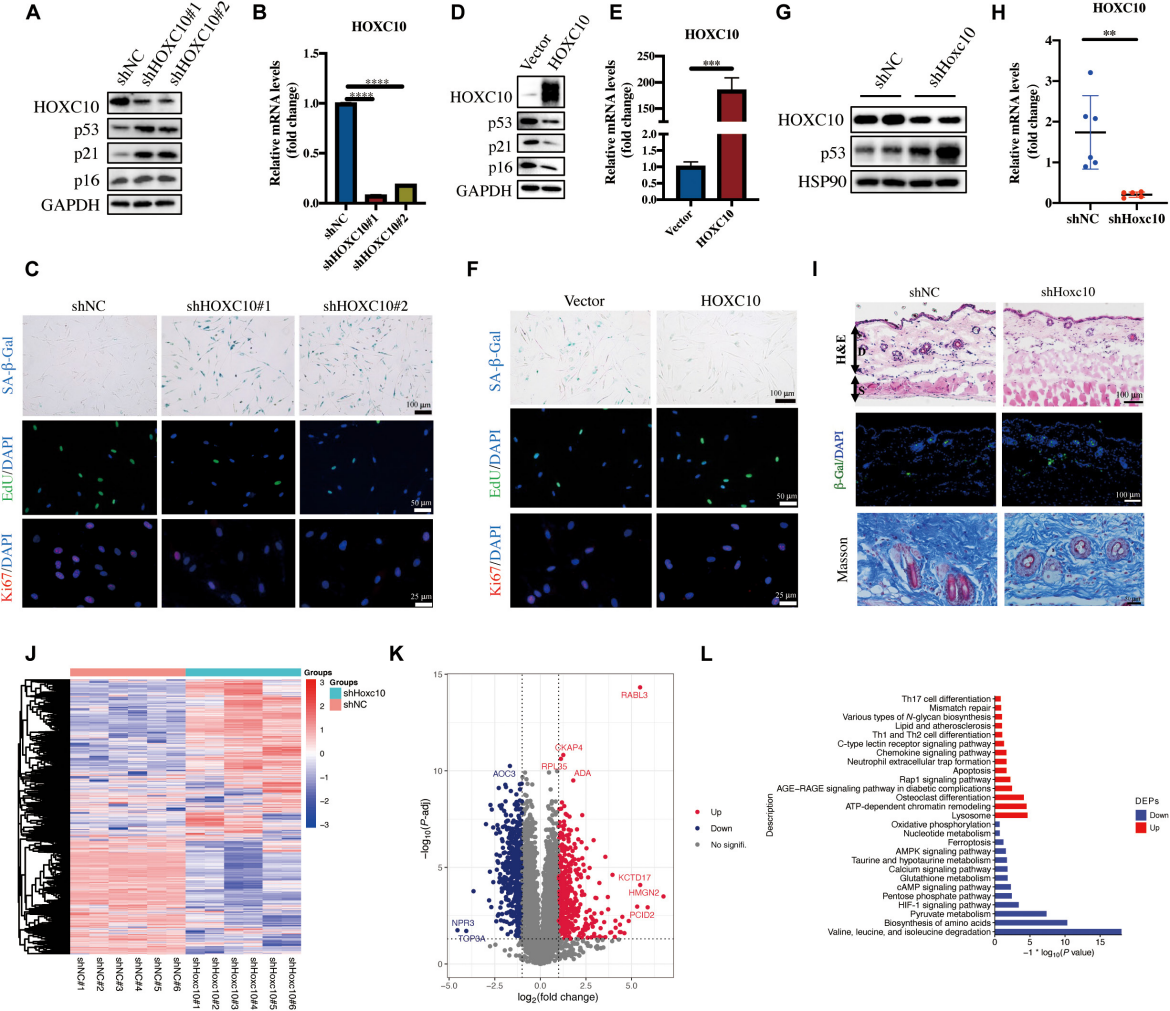
Down-regulated HOXC10 promotes cellular senescence and accelerates skin aging. Young-passage HDFs (population doublings [PDs] < 10) were infected with shHOXC10 or negative control shNC. The shHOXC10 down-regulated the protein levels of HOXC10, p53, p21, p16, and lamin B by western blotting (A) and the mRNA expression of HOXC10 by qRT-PCR (B). (C) Immunofluorescence staining of Ki67, 5-ethynyl-2′-deoxyuridine (EdU), and γ-H2A.X and senescence-associated β-galactosidase (SA-β-Gal) staining in HDFs upon short hairpin RNA (shRNA)-mediated knockdown of HOXC10. Senescent HDFs (PDs > 35) were transfected with HOXC10 or the vector. The HOXC10 up-regulated the protein levels of HOXC10, p53, p21, p16, and lamin B as shown by western blotting (D); the mRNA expression of HOXC10 by qRT-PCR (E). (F) Immunofluorescence staining of Ki67 and EdU and SA-β-Gal staining in HDFs upon overexpression of HOXC10. The protein (G) and mRNA (H) expression in the shHOXC10 skin of mice (*n* = 6 mice per group, mean age 2 months). (I) Representative images of hematoxylin and eosin (H&E) staining, β-Gal immunofluorescence staining, and Masson staining in skin tissues. (J) The heatmap of differentially expressed proteins (DEPs). (K) The volcano plot revealed the DEPs between the shNC and shHOXC10 groups. (L) The Kyoto Encyclopedia of Genes and Genomes (KEGG) enrichment analysis of DEPs. Pathways highlighted as aging related include the advanced glycation end products–receptor of advanced glycation end products (AGE–RAGE) signaling pathway in diabetic complications, apoptosis, T helper 1 (Th1) and Th2 cell differentiation, Th17 cell differentiation, and ras-associated protein 1 (Rap1) signaling pathway. The data are shown as mean ± SEM; **P ≤ 0.01; ***P ≤ 0.001; ****P ≤ 0.0001. DAPI, 4′,6-diamidino-2-phenylindole.

To explore the role of HOXC10 in fibroblast senescence, we employed lentiviral vectors to silence HOXC10 in young HDFs (population doublings [PDs] < 10) and to overexpress HOXC10 in old HDFs (PDs > 35). Both shHOXC10#1 and shHOXC10#2 effectively reduced the messenger RNA and protein levels of HOXC10 in HDFs (Fig. 2A and B). Silencing HOXC10 led to the up-regulation of senescence markers, including p53, p21, and p16 (Fig. 2A and Fig. S3A), as well as an increase in senescence-associated β-galactosidase (SA-β-Gal) staining (Fig. 2C). Data from Cell Counting Kit-8 (CCK-8), 5-ethynyl-2′-deoxyuridine (EdU), and Ki67 experiments showed that deficiency of HOXC10 significantly inhibited HDF viability and proliferation (Fig. 2C and Fig. S3B and C). The expression levels of BCL-2 and Bax indicated that HOXC10 depletion did not affect apoptosis (Fig. S3D). HOXC10 depletion also increased the expression of SASPs, such as IL-1α, FGF2, CXCL3, IL-1β, TNF-α, and MMP3 (Fig. S3E). In contrast, overexpressed HOXC10 attenuated senescence-associated phenotypes in D-gal-induced cell senescence (Fig. S3F) and in replicative senescence (Fig. 2D to F and Fig. S3G and H).

We then evaluated the effect of HOXC10 depletion on skin aging using a mouse model. Short hairpin RNA (shRNA) lentiviruses (*shNC* and *shHoxc10*) were intradermally injected into the back skin of mice over the course of 8 weeks. Firstly, we confirmed the significant knockdown of *Hoxc10* expression in the skin by western blot and qRT-PCR (Fig. 2G and H). Knockdown of *Hoxc10* promoted the skin aging process in mice, including the increased levels of aging markers and the histological senescence-like changes in the skin. Compared with the *shNC* group, *Hoxc10* depletion led to an increase in the expression of senescence markers, including p53, p16, and β-Gal (Fig. S4A and B and Fig. 2I). Hematoxylin and eosin (H&E) staining and Masson’s trichrome staining revealed that the epidermis and dermis were significantly thinner in the *Hoxc10*-depleted group, with thicker subdermal tissue, mimicking the characteristics of older mice (Fig. 2I and Fig. S4C). Furthermore, Masson’s trichrome staining indicated that collagen fibers in the dermis of the *Hoxc10*-depleted group were thinner and more loosely organized (Fig. 2I). Additionally, the *Hoxc10* depletion group exhibited increased levels of SASP, including *Il6*, *Il1b*, *Cxcl12*, *Tnf*, *Mmp3*, and *Fgf2* (Fig. S4D). HDFs with knockdown of *Hoxc10* were collected for proteome detection and Kyoto Encyclopedia of Genes and Genomes (KEGG) analysis, which showed that aging-related pathways were up-regulated after *Hoxc10* depletion (Fig. 2J to L). These findings underscore the pivotal role of HOXC10 in regulating HDF senescence, with its down-regulation promoting the acceleration of skin aging.

### FZD6 is the direct target of HOXC10

**Fig. 3.**
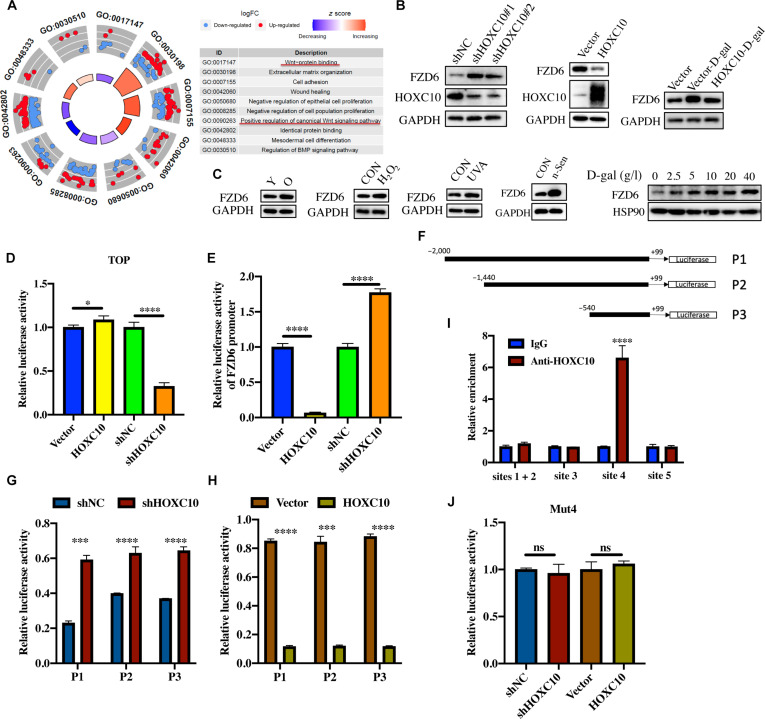
HOXC10 regulates the promoter activity of frizzled 6 (FZD6) in the Wnt/β-catenin pathway. (A) The enrichment analysis of Gene Ontology (GO) showing the pathways enriched for differentially expressed genes identified between the shNC and shHOXC10 cells. (B) The expression of FZD6 was examined by western blotting in HDFs following the knockdown or overexpression of HOXC10. (C) The expression of FZD6 in aged HDFs was analyzed by western blotting. (D) TOPflash (reporter gene plasmid) detects the level of β-catenin-mediated T cell factor/lymphoid enhancer factor (TCF/LEF) transcriptional activity in the Wnt signaling pathway. (E) The FZD6 promoter luciferase reporter plasmids and pRL-TK plasmids were transfected into the empty vector-transduced, HOXC10-transduced, control shRNA-transduced, and the shHOXC10-transduced glioma cells. Twenty-four hours later, the luciferase signal was examined. (F) Schematic illustration of the cloned fragments of the human FZD6 promoter. (G) Luciferase activity of FZD6 promoter fragments in shRNA control vector cells and HOXC10-silenced cells. (H) Luciferase activity of FZD6 promoter fragments in control vector cells and HOXC10-overexpressing cells. (I) Chromatin immunoprecipitation (ChIP) assays were performed using an anti-HOXC10 antibody to screen HOXC10-binding sites in fragment P3 of the FZD6 promoter. Immunoglobulin G (IgG) was used as a negative control. (J) Relative luciferase activity of each group after mutation site 4. Data are shown as mean ± SEM. *P < 0.05; ***P < 0.001. UVA, ultraviolet A.

To further explore the mechanisms by which HOXC10 influences aging, we conducted transcriptome analysis on both control and HOXC10-knockdown HDFs (Fig. S5A and B). This analysis identified a total of 1,092 differentially expressed genes (DEGs) (Supplementary Material 2). Enrichment analysis revealed that these DEGs were significantly linked to several biological pathways, including Wnt signaling, cell adhesion, wound healing, and extracellular matrix remodeling (Fig. 3A). Given the well-established role of Wnt signaling in modulating aging processes [7], we focused on identifying the potential target genes of HOXC10 within the DEGs associated with the Wnt pathway.

Subsequently, we used the Genome Browser website to predict the promoter regions of Wnt pathway-related DEGs that have potential binding sites for HOXC10, which include FOXO1, PYGO1, and FZD6. Further, qRT-PCR results showed that FZD6 is the most likely target gene of HOXC10 (Fig. S5C). We further evaluated the effect of HOXC10 on the expression of FZD6 and found that HOXC10 reversely regulates the expression of FZD6 (Fig. S5C). Analysis of skin single-cell transcriptomic data demonstrated an elevated expression level of FZD6 in aged skin compared to that in young ones (Fig. S5D). In line with this, western blot analysis demonstrated that knocking down HOXC10 significantly increased FZD6 expression, while overexpressing HOXC10 reduced FZD6 levels (Fig. 3B). In addition, FZD6 showed high expression levels in replicative senescent HDFs and senescent HDFs induced by H_2_O_2_, ultraviolet A, n-Sen, and D-gal (Fig. 3C). FZD6 is an important receptor for Wnt molecules and has been reported to play a regulatory role in the Wnt signaling pathway [26,27]. To confirm the regulatory relationship between HOXC10 and the Wnt pathway, we performed a luciferase assay using the TOPflash reporter and pRL-TK as an internal control. The results demonstrated that overexpression of HOXC10 enhanced while knockdown of HOXC10 decreased Wnt/β-catenin pathway activity, further supporting the role of HOXC10 in modulating the Wnt signaling pathway (Fig. 3D).

To further investigate the mechanisms by which HOXC10 regulates FZD6, we conducted a luciferase reporter gene assay. The results showed that overexpression of HOXC10 inhibited luciferase activity in the FZD6 promoter, while silencing HOXC10 led to an enhancement of luciferase activity (Fig. 3E). Subsequently, we used the JASPER website to examine the FZD6 promoter sequence, identifying potential HOXC10-binding motifs (Table S2). To pinpoint the exact binding site, we divided the promoter region into 3 segments based on the location of these motifs: P1 (−2,000 to +99), P2 (−1,440 to +99), and P3 (−540 to +99). These fragments were then cloned into the luciferase reporter vector pGL4.17 (Fig. 3F). The luciferase assay revealed that HOXC10 knockdown or overexpression significantly altered FZD6 promoter activity in all 3 segments (Fig. 3G and H), suggesting that the binding site for HOXC10 is likely located within the P3 segment (−540 to +99). There are 5 predicted binding sites in the P3 segment, which are defined as sites 1, 2, 3, 4, and 5 according to the direction of transcription. Further, chromatin immunoprecipitation (ChIP) and qRT-PCR experiments were used to identify possible binding sites for HOXC10 on the P3 fragment, revealing an interaction at site 4 (Fig. 3I). Next, we performed nucleotide mutations on site 4 in the P3 fragment region of the FZD6 promoter, changing ATCaataAAA to ATCgccgAAA. This mutation reversed the effect of HOXC10 overexpression or knockdown on the luciferase activity of site 4 (Fig. 3J). Taken together, these findings provide strong evidence that HOXC10 regulates FZD6 expression by binding to specific motifs in its promoter, thereby influencing its promoter activity.

### FZD6 is indispensable for HOXC10-induced Wnt/β-catenin signaling activation and senescence

**Fig. 4.**
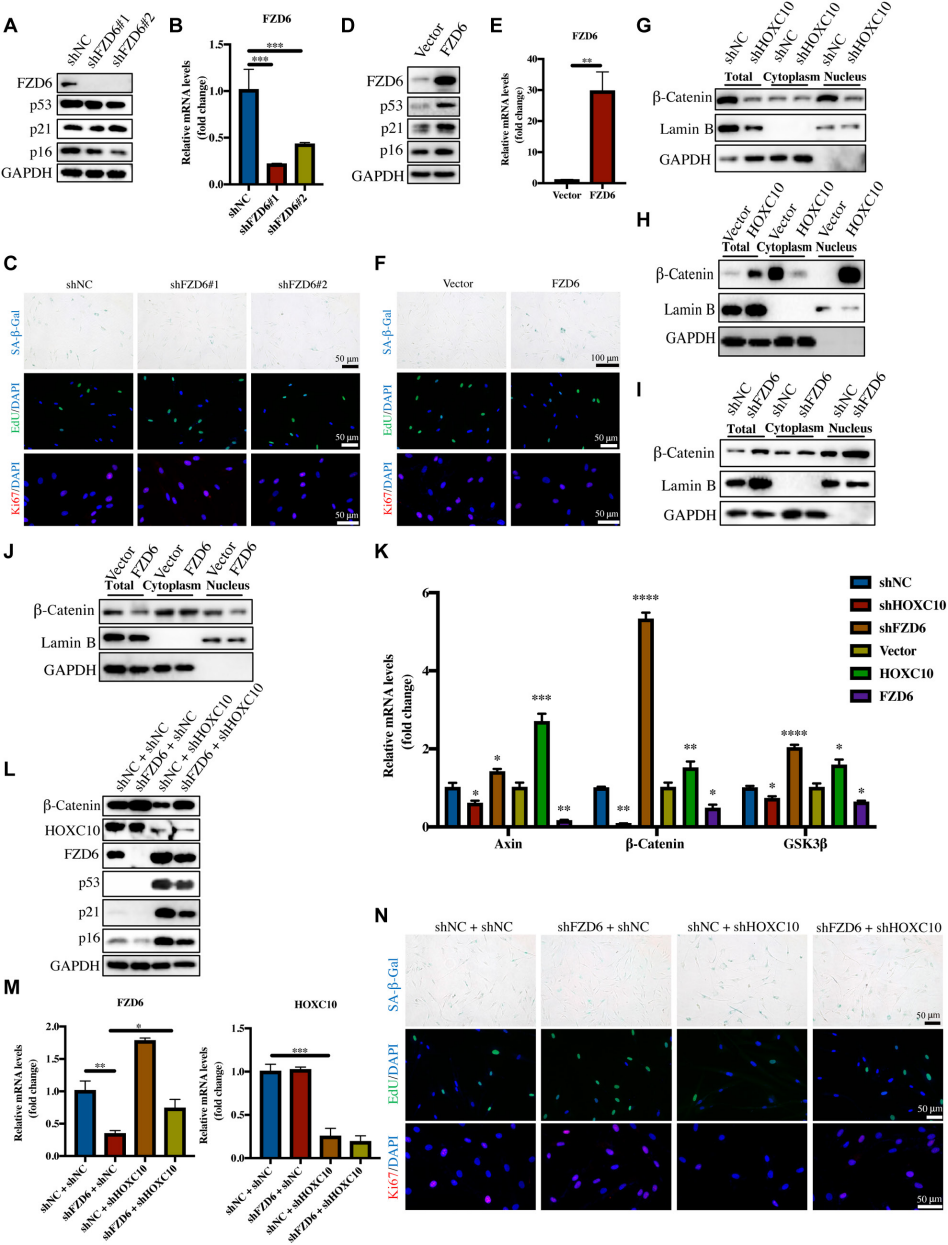
FZD6 is indispensable for HOXC10-mediated activation of Wnt/β-catenin signaling and aging progression. Senescent HDFs (PDs > 35) were infected with shFZD6 or negative control shNC. The protein levels of FZD6, p53, p21, and p16 (A) and the mRNA expression of FZD6 (B) after FZD6 knockdown. (C) Immunofluorescence staining of Ki67 and EdU and SA-β-Gal staining in HDFs upon shRNA-mediated knockdown of FZD6. Scale bars: 50 μm. Young-passage HDFs (PDs < 10) were infected with FZD6 or the vector. The protein levels of FZD6, p53, p21, and p16 (D) and the mRNA expression of FZD6 (E) after FZD6 overexpression. (F) Immunofluorescence staining of Ki67 and EdU and SA-β-Gal staining in HDFs upon overexpression of FZD6. Long scale bars: 100 μm; short scale bars: 50 μm. (G and H) Expression of the total protein, cytoplasmic protein, and nuclear protein of β-catenin after HOXC10 knockdown and overexpression. (I and J) Expression of the total protein, cytoplasmic protein, and nuclear protein of β-catenin after FZD6 knockdown and overexpression. (K) The mRNA expression of Axin, glycogen synthase kinase-3β (GSK3β), and β-catenin in HOXC10 knockdown/overexpression and FZD6 knockdown/overexpression. The rescue assay was used to assess the reversed effect of shFZD6 in shHOXC10-mediated senescence. (L) The expression of β-catenin, HOXC10, FZD6, p53, p16, and p21 was verified at the protein level. (M) The expression of HOXC10 and FZD6 was verified at the mRNA level. (N) Immunofluorescence staining of Ki67 and EdU and SA-β-Gal staining indicated that FZD6 knockdown could reverse shHOXC10-induced HDF senescence. Scale bars: 50 μm. Data are shown as mean ± SEM. *P < 0.05; **P < 0.01; ***P < 0.001; ****P < 0.0001.

To determine the roles of FZD6 in skin aging, we used lentivirus to knock down or overexpress FZD6 in HDFs (Fig. 4A, B, D, and E). After knocking down FZD6 in aged HDFs, we observed an attenuation of cellular senescence, as evidenced by the reduced levels of senescence markers and SASP (Fig. 4A to C and Fig. S6A). Moreover, overexpression of FZD6 in HDFs promoted cell senescence (Fig. 4D to F and Fig. S6B). Overexpression of FZD6 in mouse skin led to elevated p53 expression and histological features of accelerated aging, including epidermal thinning and collagen loss (Fig. S7A to D). qRT-PCR further confirmed that FZD6 up-regulation significantly increased SASP factor expression in skin (Fig. S7E).

Given the crucial roles of FZD6 in the Wnt pathway, we hypothesized that HOXC10 regulates this pathway by negatively regulating FZD6. First, we examined the effect of HOXC10 on the Wnt pathway. A key event in Wnt/β-catenin pathway activation is the translocation of β-catenin from the cytoplasm to the nucleus. We assessed whether HOXC10 affects this translocation process. Our findings indicated that silencing HOXC10 inhibited Wnt signaling, as evidenced by reduced total β-catenin levels and its diminished nuclear translocation in HDFs (Fig. 4G). Conversely, as shown in Fig. 4H, overexpression of HOXC10 activated the Wnt pathway.

We then examined the influence of FZD6 on the Wnt signaling pathway. Contrary to the effects observed with HOXC10, silencing FZD6 led to an increase in β-catenin levels in both the cytoplasm and nucleus, whereas overexpression of FZD6 inhibited Wnt/β-catenin signaling (Fig. 4I and J). To further investigate the roles of HOXC10 and FZD6 in regulating the Wnt/β-catenin pathway, we evaluated the expression of downstream genes, including Axin and glycogen synthase kinase-3β (GSK3β). Consistent with the observed changes in β-catenin, we found that Axin and GSK3β levels were reduced in HDFs with HOXC10 knockdown or FZD6 overexpression, while these proteins were up-regulated in cells with HOXC10 overexpression or FZD6 knockdown (Fig. 4K).

To ascertain the critical role of FZD6 as a mediator of HOXC10-mediated regulation of skin aging, we knocked down FZD6 in HOXC10-depleted cells (Fig. 4L and M). Wnt/β-catenin signaling was down-regulated upon HOXC10 knockdown, and this down-regulation was partially reversed by FZD6 depletion. Moreover, we observed that FZD6 depletion partially attenuated HOXC10 knockdown-induced aging (Fig. 4L and M). In addition, knockdown of FZD6 significantly increased cell proliferation and reduced SASP in HOXC10-depleted HDFs (Fig. 4N and Fig. S8). Taken together, these results strongly suggest that FZD6 is essential for HOXC10-mediated inhibition of Wnt/β-catenin signaling and HDF senescence.

### SIM, a mimic drug of HOXC10, delays cellular senescence

**Fig. 5.**
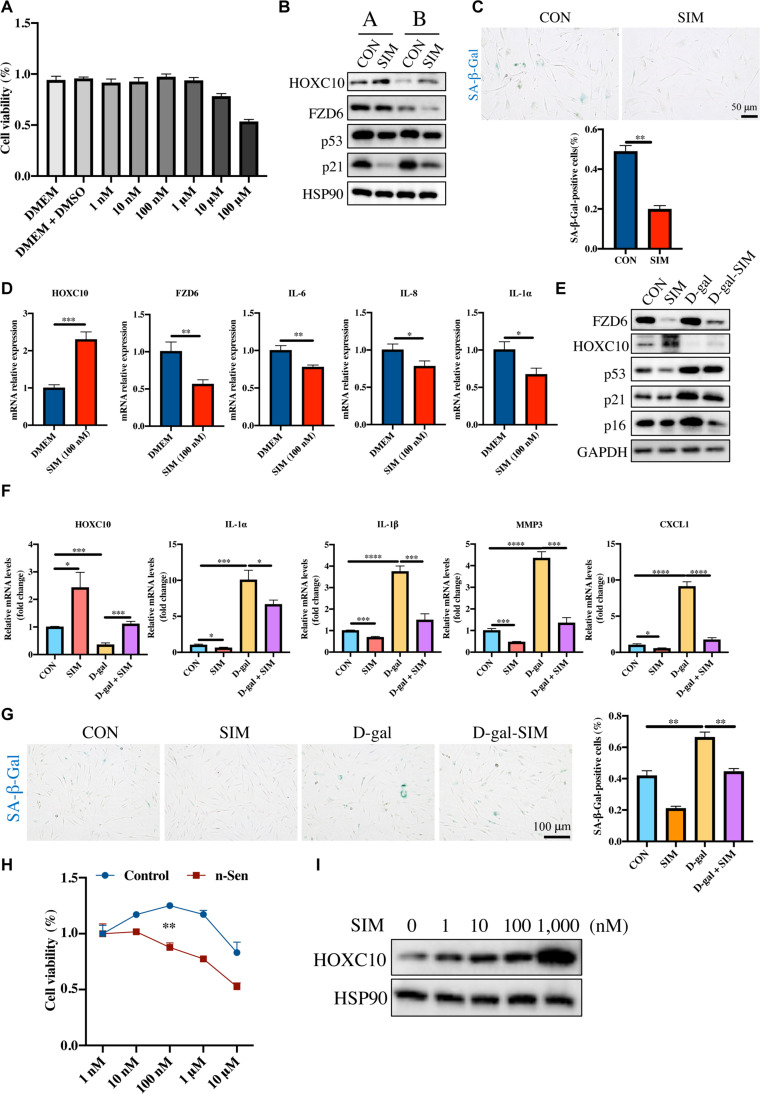
Simvastatin, a mimic drug of HOXC10, also has antiaging effect. (A) Cell viability was measured at different concentrations of simvastatin. (B to D) The A and B individuals were passaged from about 15 generations to about 25 generations, with or without simvastatin. (B) The expression of HOXC10, FZD6, p53, and p21 was verified at the protein level. (C) Representative images (up) and statistics (down) of SA-β-Gal staining. Scale bar: 50 μm. (D) The expression of HOXC10, FZD6, IL-6, IL-8, and IL-1α was verified at the mRNA level. (E to G) The D-gal aging model was induced with or without simvastatin treatment. (E) The expression of HOXC10, FZD6, p53, p16, and p21 was verified at the protein level. (F) The expression of HOXC10, IL-1β, MMP3, CXCL1, and IL-1α was verified at the mRNA level. (G) Representative images (left) and statistics (right) of SA-β-Gal staining. Scale bar: 100 μm. (H) Young and n-Sen cells were treated with different concentrations of simvastatin, and cell viability was measured. (I) The protein level expression of HOXC10 was detected 48 h after cells were treated with different concentrations of simvastatin. All results are representative results from at least 3 repeated independent experiments. Data are shown as mean ± SEM. *P < 0.05; **P < 0.01; ***P < 0.001; ****P < 0.0001. DMEM, Dulbecco’s modified Eagle’s medium; DMSO, dimethyl sulfoxide; SIM, simvastatin.

Considering the antiaging role of HOXC10, we searched for drugs that could mimic the functional targets of HOXC10 by using the Connectivity Map, and SIM was chosen for further analysis following a comprehensive evaluation of its efficacy and potential side effects. We tested the cytotoxic effect of SIM in HDFs even though it is widely used in clinical practice and is recognized as a relatively safe drug. SIM had no obvious cytotoxicity to HDFs until the concentration was increased to 1 μM (Fig. 5A). Interestingly, the cell viability of HDFs was slightly increased with 100 nM SIM. Therefore, 100 nM was selected for further analysis.

Next, we investigated whether SIM affects aging in HDFs. We treated HDFs from various individuals with SIM and subsequently passaged the cells. Our results revealed that SIM significantly reduced the levels of the aging markers p53, p21, p16, and SASP, as well as SA-β-Gal staining (Fig. 5B to D). It is worth mentioning that the HOXC10 was up-regulated and FZD6 was down-regulated by SIM treatment (Fig. 5B and D). SIM also mitigated D-gal-induced senescence in HDFs, as demonstrated by reductions in senescence marker and SASP levels, along with decreased SA-β-Gal staining (Fig. 5E to G). Furthermore, SIM reversed D-gal-induced the down-regulation of HOXC10 and up-regulation of FZD6 (Fig. 5E and F).

To further assess whether SIM effectively clears senescent cells, we used nutlin-3a (n-Sen) to induce cell senescence. This method can obtain nearly 100% senescent cells [25]. We found that SIM inhibited the viability of n-Sen-induced senescent HDFs in a dose-dependent manner, particularly at concentrations exceeding 10 nM (Fig. 5H). This suggests that it may have the potential to eliminate senescent cells. Moreover, with the increase in SIM concentration, the expression of HOXC10 in HDFs gradually increased (Fig. 5I). These results demonstrate that SIM holds promise in delaying aging, potentially through its action on the HOXC10–FZD6 axis.

### SIM delays D-gal-induced aging in mice

**Fig. 6.**
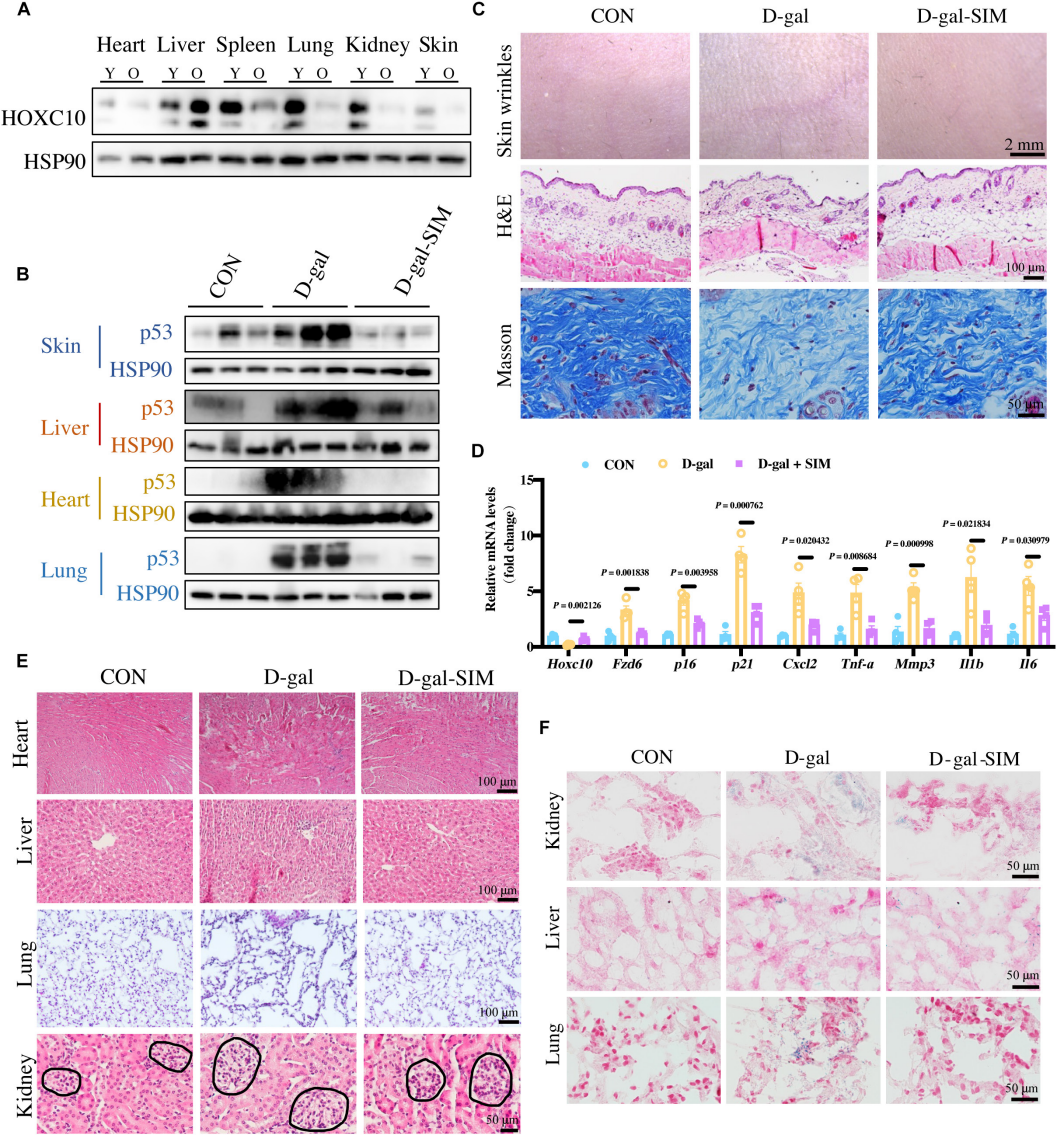
Simvastatin treatment can delay the systemic senescence induced by D-gal. (A) The protein expression levels of HOXC10 in different tissues of young and old mice (*n* = 6 mice per group). Two-month-old mice were divided into 3 groups: wild-type normal control group (CON), wild-type D-gal model group (D-gal), and wild-type D-gal and simvastatin treatment group (D-gal-SIM) (*n* = 6 mice per group). (B) The protein expression levels of p53 in different groups of mouse tissues (*n* = 6 mice per group). (C) Representative images of skin texture, H&E staining, and Masson staining of mouse skin from different groups. (D) The mRNA expression in the skin of mice in different groups. (E) Representative images of H&E staining of heart, liver, kidney, lung from different groups. Long scale bars: 100 μm; short scale bar: 50 μm. (F) Representative images of SA-β-Gal staining of the kidneys, livers, and lungs from different groups. Scale bars: 50 μm.

We investigated the expression of HOXC10 in various organs of naturally aging mice and discovered that its expression was significantly reduced in several aging tissues, including the heart, spleen, lung, kidney, and skin (Fig. 6A). The down-regulation of HOXC10 seems to be a widespread characteristic of aging across multiple organs, suggesting that HOXC10 not only is implicated in skin aging but also plays a crucial role in the aging processes of other organs throughout the body.

To further explore the effects of SIM on systemic aging, we employed a D-gal-induced aging mouse model. C57BL/6J mice, aged 8 weeks, were administered D-gal at a dose of 500 mg/kg daily for 8 weeks. During this period, the mice in the D-gal-SIM group were also treated daily with SIM (2 mg/kg) via gavage. Notably, SIM treatment demonstrated significant potential in mitigating systemic aging, effectively reducing the D-gal-induced increase in p53 levels in various organs, including the skin, liver, heart, and lungs, despite some individual variability (Fig. 6B). We first focused on the impact of SIM on skin aging. D-gal-induced skin senescence was evaluated through skin texture imaging, H&E staining, and Masson’s trichrome staining (Fig. 6C). Compared to the D-gal group, SIM treatment (D-gal-SIM group) notably alleviated the effects of D-gal-induced skin aging, as evidenced by a reduction in skin wrinkles, thickening of the dermis, and an increase in collagen fiber content (Fig. 6C). Furthermore, SIM treatment significantly diminished the levels of senescence markers induced by D-gal (Fig. 6D). In line with the in vitro findings, SIM also reversed the alterations in HOXC10 and FZD6 expression in the skin of treated mice (Fig. 6D). Further evaluation of organ health was conducted using H&E staining. In D-gal-induced aging mice, myocardial fibers appeared disorganized, hepatic lobules exhibited structural damage with inflammatory cell infiltration, and glomerular and alveolar regions were enlarged. These pathological changes were notably reduced following SIM treatment (Fig. 6E). Additionally, compared to the D-gal group, SIM-treated mice showed significantly lower SA-β-Gal activity in the kidney, liver, and lungs (Fig. 6F). Together, these findings suggest that SIM offers a protective effect against D-gal-induced aging in mice.

## Discussion

Aging is characterized by the gradual decline in the physiological functions of tissues and organs, which considerably increases the risk of chronic diseases and negatively affects overall health. The skin, as the body’s external barrier, is in constant interaction with the environment and serves as a link to the nervous, immune, and circulatory systems. Therefore, skin aging plays a pivotal role in the broader context of systemic aging and is considered a key factor in the overall aging of the body [2]. In this study, we identified HOXC10 as a critical regulator of aging. HOXC10 directly targets and inhibits the expression of FZD6, thereby activating the Wnt/β-catenin signaling pathway, which contributes to the delay of the aging process.

There is accumulating evidence underscoring the pivotal role of transcription factors in the complex regulatory networks that govern aging [28]. In this study, we observed an important down-regulation of HOXC10 in aging HDFs and various organs, indicating that HOXC10 may serve as a novel marker of organ aging. Previous research has shown that HOXC10 is overexpressed in numerous tumors [21–23], where it promotes tumor cell proliferation through the activation of signaling pathways such as the MAPK and ERBB3/PI3K/AKT axes [29–31]. Additionally, Guerra et al. [32] demonstrated that overexpression of HOXC10 increases the expression of genes involved in DNA replication, such as E2F and pre-replicative complexes, which are crucial for cell cycle progression. Here, we demonstrated that the depletion of HOXC10 plays an important role in the induction of HDF senescence and the acceleration of skin aging. Telomere shortening, mitochondrial dysfunction, and DNA damage are well-recognized hallmarks of aging, and previous studies have suggested that certain HOX family members may influence these processes through transcriptional regulation of genomic-stability-related genes [21,33]. Although our study primarily focused on cellular senescence, it is possible that HOXC10 may also exert antiaging effects by modulating these pathways, which will be further explored in future research.

The Wnt signaling pathway has garnered notable attention due to its crucial role in various cellular processes and its involvement in numerous human diseases. Several studies have highlighted its beneficial effects in promoting health and delaying aging [6,34]. Modulating and activating Wnt signaling has shown considerable therapeutic promise in age-related conditions such as Alzheimer’s disease and osteoporosis, as well as in delaying senescence in intestinal stem cells [7,35–39]. Moreover, activation of the Wnt/β-catenin pathway in the skin not only protects against ultraviolet B-induced photoaging but also accelerates skin wound healing [40,41]. In our study, we found that HOXC10 up-regulates the Wnt/β-catenin signaling pathway, suggesting that this could be the underlying mechanism through which HOXC10 influences cellular senescence.

The FZD proteins, members of the “frizzled” gene family, act as receptors for Wnt ligands in mammals. All 10 subtypes of the FZD family (FZD1 to FZD10) participate in both classical and nonclassical Wnt signaling pathways [42,43]. Wnt ligands bind to various FZD receptors, forming complexes that can either activate or inhibit downstream signaling pathways, such as Wnt/β-catenin, Wnt/PCP, and Wnt/Ca^2+^ [44,45]. In this study, we observed that HOXC10 negatively regulates the expression of FZD6 by inhibiting its promoter activity. FZD6 has previously been identified as a negative regulator of the Wnt/β-catenin pathway [26,27]. For example, Golan et al. [27] demonstrated that FZD6 activates TAK1 and NLK kinases, which subsequently inhibit the Wnt/β-catenin signaling-dependent transcription of target genes. Additionally, FZD6 expression has been shown to increase in the aging thymus of mice [46]. Another study suggested that knockdown of FZD6 promotes the progression of the cell cycle in thymus epithelial cells [47]. Despite these observations, the precise role of FZD6 in aging remains unclear. In our study, we identified FZD6 as a downstream target of HOXC10, where it functions to repress Wnt/β-catenin signaling and facilitate cellular senescence.

Recently, the development of antiaging drugs targeting aging regulators has become a prominent focus of scientific investigation. By using the Connectivity Map method [48], we identified SIM as a mimic HOXC10 with a potential antiaging effect. SIM is widely used clinically, with good tolerance and high safety. Statins are recognized for being beneficial in lowering blood lipids and providing anti-inflammatory, antioxidation, and antiatherosclerosis effects [49]. Increasing evidence suggests that statins also possess antiaging potential [50]. For example, atorvastatin, pitavastatin, and pravastatin have been found to up-regulate the expression of the antiaging gene klotho [51,52]. In animal models, long-term atorvastatin treatment can improve age-related endothelial dysfunction and reverse the aging manifestations of the heart [53,54]. Moreover, the combination of pravastatin and aminobisphosphonates markedly improves aging-like phenotypes and prolongs the lifespan of prematurely aged mice [55]. SIM has been reported to alleviate aging in chondrocytes, vascular endothelial cells, and fetal membranes [56–58], which may be attributed to its antioxidant and anti-inflammatory effects [59,60]. Consistently, we found that SIM noticeably ameliorates fibroblast senescence and organ aging in mice. Mechanically, SIM delays aging not only by attenuating cellular senescence partly by up-regulating HOXC10 and reducing the expression of FZD6 (Figs. S9 and S10) but also by eliminating senescent cells.

This study has several limitations. First, while SIM was effective in alleviating skin aging phenotypes in our D-gal-induced mouse model, the potential systemic side effects of oral statin administration—such as hepatotoxicity, myopathy, and alterations in lipid metabolism—should be considered before clinical translation. Future studies using topical formulations or targeted delivery systems may help reduce systemic exposure and improve safety. Second, while our current study primarily focused on HOXC10 knockdown in vivo, we acknowledge the need for complementary gain-of-function approaches. In future work, we plan to test HOXC10 overexpression using skin viral delivery and transgenic mouse models to evaluate whether elevated HOXC10 can delay epidermal thinning, reduce senescence-associated phenotypes, and modulate additional hallmarks of aging. Such experiments will not only complement our present findings but also provide deeper insights into the therapeutic potential of manipulating HOXC10 in aging- and age-related diseases. Third, the D-gal model was chosen for its reproducibility, efficiency, and ability to mimic oxidative stress, inflammation, and senescence-associated phenotypes. However, it does not reflect all features of natural aging, particularly genomic instability, telomere attrition, and stem cell exhaustion. Models such as chronic low-dose radiation or naturally aged mice capture these long-term processes more faithfully but are more time and resource intensive. Thus, while the D-gal model is valuable for mechanistic studies and drug screening, validation in naturally aged animals will be necessary to confirm whether HOXC10 and SIM act across the full spectrum of aging hallmarks.

In summary, our study has elucidated the antiaging role of HOXC10, highlighting its regulatory impact on the FZD6/Wnt/β-catenin signaling pathway. Furthermore, we identified SIM, a mimic of HOXC10, as a promising therapeutic strategy for delaying the aging process.

## Materials and Methods

### Animal models

Male C57BL/6J rodents (6 to 8 weeks old) were obtained from Slack Biotechnology Corporation and maintained in the animal research facility of Xiangya Medical Center. All animal experiments were performed following the ethical standards established by the National Institutes of Health for laboratory animal welfare. The animals were acclimatized under standardized conditions, including maintained ambient temperature (22 ± 2 °C), 60% humidity, controlled photoperiod (12-h light/dark), and sound pressure below 60 dB. Nutritional requirements were met through ad libitum access to standard chow and purified water.

Recombinant lentiviral constructs targeting HOXC10 gene silencing or corresponding empty vector controls were reconstituted in phosphate-buffered saline (PBS; Gibco, Thermo Fisher Scientific, Massachusetts, USA) to achieve a final titer of 1 × 10^6^ transducing units per 50-μl aliquot. The viral suspension was administered through precise intradermal microinjections across the murine dorsal region. This intervention protocol persisted for 8 consecutive weeks. Terminal anesthesia was induced 24-h post-final injection using sodium pentobarbital (80 mg/kg body weight).

Genetically unmodified subjects were stratified into 3 experimental cohorts: untreated controls (CON), D-gal-induced aging models (D-gal), and D-gal plus SIM intervention group (D-gal-SIM). The modeling agent (D-gal) was prepared in physiological saline (0.9% NaCl) and delivered via daily intraperitoneal injections (500 mg/kg/d) throughout the 8-week study period. The D-gal-SIM cohort received concurrent oral SIM therapy (2 mg/kg/d) alongside D-gal administration. Final experimental procedures were conducted under pentobarbital sodium anesthesia (80 mg/kg) 24 h after treatment cessation.

### Investigation of human cutaneous specimens

This research utilized dermal biopsies collected from participants categorized into 2 demographic cohorts: younger individuals (*n* = 23; aged 16 to 28 years; mean ± SD: 23.1 ± 3.4) and older adults (*n* = 31; aged 53 to 74 years; mean ± SD: 62.1 ± 5.7).

### Cell culture

Primary fibroblasts were derived from clinically discarded foreskin specimens collected from male donors aged 5 to 35 years following routine circumcision. Prior to sample collection, written consent forms were signed by all donors, and the study protocol was reviewed and approved by the Ethics Committee at Xiangya Hospital.

Cell cultures were established using Dulbecco’s modified Eagle’s medium (DMEM; Thermo Fisher Scientific) enriched with 10% fetal bovine serum (Thermo Fisher Scientific) and a 1% antibiotic–antimycotic solution (penicillin–streptomycin; Thermo Fisher Scientific). For comparative studies, 293T human embryonic kidney cells (ATCC CRL-3216) were cultured in parallel. All cell lines were grown in a humidified incubator at 37 °C with 5% carbon dioxide and 95% relative humidity.

For experimental stratification, fibroblast cultures were categorized based on passage number: early-passage cells (<15 PDs) were designated as young HDFs, whereas late-passage cells (>35 doublings) were classified as senescent fibroblasts.

### Cellular senescence model of HDFs

#### Replicative senescence model

To induce replicative senescence, early-passage fibroblasts (passages 5 to 8) were continuously subcultured until reaching proliferative exhaustion (typically passages 35 to 45). Senescence status was confirmed by assessing β-galactosidase activity and proliferation markers. This model represents physiologically relevant aging in vitro.

#### Oxidative-stress-induced senescence

To trigger premature senescence via oxidative stress, cultured cells were exposed to 1 mM hydrogen peroxide (H_2_O_2_) for a 2-h period. Following this treatment, the oxidative medium was removed and substituted with complete DMEM for subsequent cultivation. Parallel control cultures were processed identically but without H_2_O_2_ administration. After 48 h of recovery, all cellular samples were harvested for analysis.

#### Pharmacologically induced senescence

For drug-mediated senescence induction, cell cycle synchronization was initially achieved through 24-h incubation with 9 μM RO3306 (Selleck Chemicals), arresting cells in the G2 phase. This was followed by an 8-h combined treatment with both RO3306 (9 μM) and nutlin-3a (5 μM; Selleck Chemicals), subsequently transitioning to nutlin-3a alone (5 μM) for 90 min. To selectively eliminate cycling cells, cultures were then maintained in 100 nM BI-2536 for 9 consecutive days, followed by an equivalent period in standard growth medium.

#### D-gal-mediated senescence

To establish D-gal-induced senescence, cellular populations were incubated for 5 d with increasing concentrations of D-gal (0 to 40 g/l). Dose–response evaluation revealed 20 g/l as the most effective concentration for consistent senescence induction, which was consequently employed for subsequent experimental procedures.

### Plasmid construction, transfection, and infection

The complete coding sequences of human HOXC10 and FZD6 were cloned into lentiviral transfer vectors to generate recombinant lentiviral particles (LV-HOXC10/LV-FZD6). An empty vector construct (LV-NC) served as the negative control. For gene silencing experiments, specific shRNA sequences against HOXC10/FZD6 were designed and incorporated into lentiviral vectors to produce shRNA-expressing viruses (LV-shHOXC10/LV-shFZD6), with scrambled shRNA (LV-shNC) as the control. Viral particles were produced by co-transfecting 293T cells with the transfer plasmids and packaging vectors using the FuGENE HD transfection system (Promega). Culture supernatants containing viral particles were harvested at 48- and 72-h time points post-transfection and then clarified through 0.45-μm membrane filtration (Fisher Scientific). Viral concentrates were obtained through polyethylene glycol (PEG8000) precipitation and subsequently reconstituted in 100 μl of PBS. Transduced cells were selected with puromycin (0.5 μg/ml) beginning 48 h after infection, with antibiotic selection maintained for 10 d to ensure stable integration. This process yielded populations stably overexpressing target genes or expressing target-specific shRNAs. For animal studies, lentiviral suspensions containing 1 × 10^6^ transducing units were administered via multiple intradermal injections across the dorsal skin region, including both central and peripheral areas.

### H&E staining

Paraffin-embedded tissue specimens underwent initial processing by immersion in xylene to remove the embedding medium, followed by sequential hydration in progressively diluted ethanol baths. Nuclear staining was performed by incubating sections in hematoxylin solution for 8 min, with subsequent differentiation achieved through brief exposure (10 s) to acid–alcohol (1% HCl in 70% ethanol). This step was followed by bluing to enhance nuclear contrast. Cytoplasmic counterstaining was accomplished using eosin solution for a 5-min duration. Following staining completion, specimens were progressively dehydrated in ascending ethanol concentrations, cleared in xylene, and permanently preserved under coverslips using neutral balsam mounting medium.

### TOPflash/FOPflash report analysis

To investigate the functional activation of the canonical Wnt/β-catenin signaling cascade, we employed a dual-reporter gene assay system consisting of TOPflash (containing wild-type T cell factor/lymphoid enhancer factor binding sites) and its mutant control FOPflash. For these experiments, 293T human embryonic kidney cells were plated into 24-well culture dishes and maintained until achieving approximately 50% confluence, at which point transfection procedures were initiated. The experimental setup involved co-transfection of 3 plasmid constructs: either HOXC10 knockdown (shHOXC10) or overexpression (PLVX-HOXC10) vectors and the β-catenin-responsive reporter plasmid (TOPflash) or its mutant control (FOPflash) (Beyotime Biotechnology, Shanghai), along with the pRL-TK *Renilla* luciferase normalization vector. Following a 24-h incubation period post-transfection, cellular lysates were prepared and analyzed using the Dual-Luciferase Reporter Assay Kit (Promega, E1910) according to the manufacturer’s protocol. Luminescence signals were quantified using a multimode microplate reader (PerkinElmer EnSpire platform), with firefly luciferase activity normalized to *Renilla* values for each sample.

### Luciferase reporter assay

To investigate transcriptional regulation, 293T cells were seeded into 96-well culture plates at an initial concentration of 10,000 cells per well and maintained under standard culture conditions for 24 h before genetic manipulation. Transfection procedures were performed employing Lipofectamine 2000 reagent (Thermo Fisher Scientific, Waltham, MA), with the following DNA constructs: 250 ng of HOXC10 expression plasmid, 50 ng of pGL4.17 reporter vector harboring the FZD6 promoter region, and 0.25 ng of pRL-TK plasmid encoding *Renilla* luciferase as an internal control. Following a 24-h incubation period post-transfection, cellular extracts were prepared and analyzed using the Dual-Luciferase Reporter Assay Kit (Promega Corporation, Beijing). Luminescence measurements were conducted with firefly luciferase signals being standardized against *Renilla* values for each experimental condition. Final results were calculated as normalized ratios and presented as percentage values compared to baseline control measurements.

### ChIP assay

The ChIP experiments were performed according to the standardized procedures provided with the Pierce Sepharose ChIP Kit (Thermo Fisher Scientific, catalog no. 26156). Initially, HDFs that had been genetically modified via lentiviral transduction were subjected to formaldehyde-mediated cross-linking (1% final concentration) for 10 min at physiological temperature (37 °C). Following cellular membrane disruption, chromatin fragmentation was achieved through enzymatic digestion using micrococcal nuclease (ChIP-validated grade). For immunoprecipitation, we utilized validated antibodies targeting HOXC10 protein, with nonspecific immunoglobulin G antibodies serving as negative controls in parallel reactions. The antibody-bound chromatin complexes were isolated using the kit’s affinity matrix. Finally, quantitative real-time polymerase chain reaction (PCR) analysis was conducted to detect and quantify specific genomic regions corresponding to potential regulatory elements within the promoter sequence of interest.

### Antibodies and reagents

The primary antibodies and reagents utilized in this study were as follows:

The antibodies included HOXC10 (ab308518, Abcam), FZD6 (ab290728 and ab290743, Abcam), anti-β-catenin (ab305261, Abcam), lamin B (ab16048, Abcam), p16 (ab220800, Abcam), p21 (109520, Abcam), p53 (sc-126, Santa Cruz), HSP90 (ab203085, Abcam), CD31 (ab222783, Abcam), Ki67 (SAB5700770, Sigma), and glyceraldehyde-3-phosphate dehydrogenase (GAPDH; ab8245, Abcam). Secondary antibodies (anti-rabbit, anti-mouse, and anti-goat) were sourced from Abcam.

### qRT-PCR

Total RNA was extracted from cells or tissue samples using an appropriate RNA extraction kit (TRIzol reagent). The RNA concentration was measured using a spectrophotometer (NanoDrop). For complementary DNA synthesis, 1 μg of RNA was used as the template in a reverse transcription reaction. The reaction is carried out using a reverse transcription kit following the manufacturer’s protocol, typically involving incubation at 42 °C for 60 min and enzyme inactivation at 70 °C for 5 min. The qRT-PCR reaction mixture was prepared by combining the complementary DNA template, primers specific to the target genes, SYBR Green Master Mix, and nuclease-free water. The final volume for each reaction was typically 20 μl. The reaction mixture was loaded into a 96-well plate, and PCR was performed on a real-time PCR machine (e.g., ABI 7500). The thermal cycling conditions usually included an initial denaturation step at 95 °C for 10 min, followed by 40 cycles of amplification (denaturation at 95 °C for 15 s, annealing at the optimal primer temperature for 30 s, and extension at 72 °C for 30 s). After amplification, the fluorescence data were collected, and the relative expression levels of the target genes were calculated using the 2^−ΔΔCt^ method, with GAPDH or β-actin used as an internal control. The results could be visualized and analyzed by plotting the Ct values, and statistical analysis was performed to determine gene expression differences.

### Western blotting

Tissue and cell samples were homogenized in radioimmunoprecipitation assay buffer (containing protease inhibitors; Beyotime, ST506) via ultrasonic disruption. After 30-min incubation on ice, lysates were centrifuged (12,000 × g, 15 min, 4 °C) to remove insoluble material, retaining the supernatant for downstream applications. Nuclear extracts were prepared using NE-PER reagents (Thermo Scientific, 78833) according to the supplied protocol. Protein concentrations were determined by bicinchoninic acid (BCA) protein assay (Pierce, 23225). Aliquots (20 to 50 μg) were mixed with Laemmli sample buffer, heat-denatured, and separated on 10% polyacrylamide gels in Tris–glycine–sodium dodecyl sulfate running buffer. Proteins were transferred to polyvinylidene fluoride membranes (0.45 μm; Millipore, IPVH00010) at 100 V for 60 min. Membranes were blocked with 5% skim milk in TBST (Tris-buffered saline with 0.1% Tween-20) for 1 h at room temperature. Primary antibody incubations were performed overnight at 4 °C in blocking solution. Following TBST washes (3 × 10 min), membranes were incubated with horseradish peroxidase (HRP)-conjugated secondary antibodies (1:10,000) for 1 h at room temperature. Signals were developed using enhanced chemiluminescence and captured with a ChemiDoc imaging system (Bio-Rad).

### Cell cycle analysis

Following experimental interventions, cellular suspensions and culture media were harvested and subjected to centrifugation at 300 × g for 5 min. The pelleted cells were resuspended in binding buffer and dual-stained with fluorescein isothiocyanate-conjugated annexin V and the viability dye propidium iodide using a commercial apoptosis detection system (Beyotime Institute of Biotechnology). To maintain fluorophore stability, the labeling procedure was carried out under light-protected conditions at ambient temperature (25 °C) for 30 min. Quantitative evaluation of apoptotic populations was performed by flow cytometric analysis (BD FACSCanto II) with appropriate compensation controls.

### EdU staining

To evaluate cellular proliferation rates, actively dividing cells were pulse-labeled with 10 μM EdU for 60 to 120 min to enable thymidine analog incorporation during DNA replication. Following incubation, samples were fixed in 4% (w/v) paraformaldehyde (PFA) solution for 15 min at room temperature and subsequently permeabilized with 0.5% Triton X-100 in PBS for 10 min. A copper-catalyzed azide–alkyne cycloaddition reaction (Click-iT chemistry) was performed using a reaction mixture containing Alexa Fluor 488–azide to fluorescently tag the incorporated EdU moieties. After extensive washing with PBS to remove unbound reagents, fluorescent signals were visualized and quantified using epifluorescence microscopy (Nikon Eclipse Ti) with appropriate filter sets for the fluorophore employed.

### SA-β-Gal staining

The presence of SA-β-Gal activity was evaluated in cultured cells and tissue specimens using a commercial staining system (Cell Signaling Technology, #9860). Following the recommended protocol, samples were processed at pH 6.0 to optimize enzymatic detection. Cells exhibiting senescence characteristics demonstrated distinct blue cytoplasmic staining when visualized under bright-field microscopy. High-resolution images of SA-β-Gal-positive cells were acquired using a laser scanning confocal microscope (Zeiss LSM 880) equipped with appropriate optical filters. For cryopreserved tissue sections, an alternative fluorescence-based senescence detection approach was employed using the CellEvent Senescence Green reagent (Thermo Fisher Scientific, catalog no. C10851), following the supplier’s recommended procedures.

### RNA-seq analysis

Total RNA was isolated from HDFs with shNC, shHOXC10#1, and shHOXC10#2 using TRIzol reagent (Thermo Fisher Scientific). Bulk RNA sequencing (RNA-seq) was performed by Ouyi Biotechnology Co., Ltd. (Shanghai). Briefly, libraries were prepared using the NEBNext Ultra RNA Library Preparation Kit, which is optimized for Illumina sequencing platforms (NEB). Transcript expression was quantified and normalized using the Salmon software. Genome alignments were generated by mapping the reads to a reference genome. Subsequently, protein-coding gene expression was analyzed, and differential expression was evaluated by comparing the variation in the expression levels of protein-coding genes across the different samples.

### CCK-8 assay

HDFs were plated into 96-well culture plates at an initial density of 20,000 cells/well in complete growth medium. Cells were allowed to adhere and proliferate under standard culture conditions (37 °C and 5% CO_2_) for the duration of the experiment. At designated time points (0, 24, 48, 72, and 96 h), 10 μl of CCK-8 solution (Sigma-Aldrich, 96992) was added to each well. The plates were gently mixed and returned to the incubator for 2 h to allow formazan dye formation. Following incubation, the optical density at 450 nm was determined using a multimode microplate reader (Thermo Fisher Scientific, Multiskan GO), with a reference wavelength of 650 nm to account for background interference.

### Proteomic profiling by liquid chromatography–tandem mass spectrometry

For proteomic analysis, approximately 25 mg of mouse skin tissue was homogenized in 200 μl of lysis buffer, and proteins were collected after centrifugation at 12,000 × g for 20 min. Protein concentrations were determined using the BCA Protein Assay Kit (Thermo Fisher Scientific, USA). An aliquot of 30 μg of total protein from each sample was subjected to digestion and processing according to the manufacturer’s protocol (OmicSolution, China).

The resulting peptides were separated on a NanoVipe-C18 analytical column (25 cm × 75 μm, Thermo Fisher Scientific) coupled to a Vanquish Neo HPLC system. Mass spectrometry analysis was performed on an Orbitrap Exploris 480 instrument (Thermo Fisher Scientific, USA). Data were acquired using the data-independent acquisition method, and raw files were processed with the Spectronaut software (version 18) for peptide identification and quantification.

### Cell viability

Primary HDFs were cultured into 96-well plates (2 × 10^4^ cells/well) using DMEM supplemented with 10% fetal bovine serum. Cell growth was evaluated over a 96-h period with measurements taken at 5 time intervals (T0 to T96). At each observation time point, 10 μl of WST-8 solution (CCK-8 assay kit, Sigma-Aldrich, catalog no. 96992) was administered to each well. Following incubation (37 °C and 5% CO_2_) for 120 min, absorbance readings at 450 nm were obtained using a SpectraMax microplate reader (Molecular Devices, California). The spectrophotometric measurements were performed with appropriate blank controls, using a reference wavelength of 600 nm to correct for nonspecific absorbance. Three independent experiments were conducted with 6 technical replicates per condition.

### Immunofluorescence staining

Following experimental treatments, cultured fibroblasts in 24-well plates were immobilized with 4% PFA solution for 15 min at ambient temperature. Cellular membranes were then permeabilized using 0.1% Triton X-100 in PBS for 10 min. Nonspecific binding sites were blocked by incubating samples with 5% goat serum (100 μl/well) at 37 °C for 60 min. Primary antibodies diluted in blocking buffer were applied (100 μl/well) and allowed to bind at 4 °C overnight. After thorough PBS washing, fluorophore-conjugated secondary antibodies were introduced and incubated for 60 min at room temperature. Cell nuclei were labeled with 4′,6-diamidino-2-phenylindole (DAPI) for 5 min prior to mounting with antifade medium. After each processing step, specimens were rinsed 3 times with PBS. Fluorescence signals were documented using digital microscopy and quantified with the ImageJ analysis software.

Fresh-frozen specimens embedded in optimal cutting temperature medium (Sakura Finetek) were cryosectioned at 6-μm thickness using a refrigerated microtome (Leica CM3050S, Germany). Sections were mounted on poly-L-lysine-coated slides and air-dried for 15 min at room temperature to ensure proper adhesion. Sections were postfixed in 4% PFA in PBS for 15 min at ambient temperature. Cellular membranes were permeabilized by incubating them with 0.3% Triton X-100 in PBS for 60 min at room temperature, followed by three 5-min PBS washes. Nonspecific binding sites were blocked with 5% normal donkey serum in PBS containing 0.1% Tween-20 for 60 min at 25 °C. Primary antibodies diluted in blocking buffer were applied and incubated at 4 °C for 16 h in a humidified chamber. Following primary antibody incubation, sections were processed with fluorophore-conjugated secondary antibodies and counterstained with DAPI for nuclear visualization. Fluorescence microscopy was performed using a laser scanning confocal system (Zeiss LSM 880) equipped with appropriate excitation/emission filters for each fluorescent probe.

### Immunohistochemistry analyses

Fresh biological samples were immersed in 4% PFA (Sigma-Aldrich) for 24 h at 4 °C to ensure optimal tissue preservation. Following fixation, specimens were processed through graded ethanol series and xylene before being embedded in paraffin wax (Paraplast, Leica) using standard histological protocols. Thin tissue sections (4 to 5 μm) were obtained using a rotary microtome (RM2235, Leica) and mounted on positively charged glass slides. Sections were then subjected to deparaffinization through 2 changes of xylene (10 min each), followed by rehydration in a graded ethanol series (100% to 50%) and final equilibration in distilled water. Heat-induced epitope retrieval was conducted in preheated 10 mM sodium citrate buffer (pH 6.0) using a water bath maintained at 95 to 100 °C for 20 min. After cooling to room temperature, endogenous peroxidase activity was inhibited by treating sections with 3% hydrogen peroxide in methanol for 15 min. Nonspecific binding was minimized by incubating sections with 10% bovine serum albumin in PBS (pH 7.4) for 30 min at room temperature. Primary antibodies, diluted in antibody diluent (Dako), were applied to sections and incubated overnight at 4 °C in a humidified chamber. After 3 washes with PBS containing 0.05% Tween-20, sections were incubated with biotinylated secondary antibodies (Vector Laboratories) for 30 min at room temperature. Signal amplification was performed using a streptavidin–HRP conjugate (Thermo Scientific) for 15 min at 37 °C. Antigen localization was visualized by developing sections with the DAB substrate (Dako) for 5 to 10 min, resulting in brown-colored precipitates at antigen sites. Nuclei were counterstained with Mayer’s hematoxylin (Sigma-Aldrich) for 1 min, followed by bluing in tap water for 5 min. Stained sections were dehydrated through graded alcohols (50% to 100%), cleared in xylene, and mounted with synthetic resin (Entellan, Merck). Digital images were acquired using a laser scanning confocal microscope (LSM 900, Zeiss) with consistent ×20 objective magnification and standardized exposure settings across all samples.

### Bioinformatics data analyses

#### Single-cell transcriptome analysis

This study used single-cell RNA-seq data of GSE130973 [61]. Data processing used Cell Ranger (v2.1.0) and Seurat (v4.0) of 10X Genomics [62]. After creating the Seurat object, cells and features were filtered, thresholds were set, and log normalization was performed. The top 2,000 most variable features were selected for downstream analysis. Principal component analysis was performed to reduce data dimensionality, retaining the first 15 principal components that collectively accounted for >85% of the total variance. These principal components were subsequently used for clustering analysis with a resolution parameter of 0.4 to optimize cluster identification. Uniform manifold approximation and projection (UMAP) was implemented for 2-dimensional representation of the high-dimensional data space. Differential gene expression analysis between clusters was conducted using the FindAllMarkers function with the following thresholds: min.pct = 0.25 and logfc.threshold = 0.25. Cluster identity was determined by evaluating the average expression levels of canonical cell-type-specific marker genes. Annotation was performed using established references from published single-cell atlases. All analyses were executed in R version 4.2.2 (2022 Oct 31) using the following packages: Seurat v4.3.0 for single-cell analysis, uwot v0.1.16 for UMAP implementation, and ggplot2 v3.4.2 for visualization.

#### Transcription factor regulatory network analysis

Single-cell transcriptome data were analyzed using Python’s SCENIC (single-cell regulatory network inference and clustering). After data preprocessing, a co-expression network was established based on the expression matrix, and GENIE3 or GRNBoost was applied to identify transcription factors and target genes. RcisTarget identifies regulatory sequences, and AUCell evaluates single-cell network activity. Results are visualized as heatmaps and network plots. The analysis was performed in the Python (v3.7) environment.

#### Differential analysis and enrichment analysis

In this study, differential analysis was conducted using the limma (Linear Models for Microarray Data) package [63], with data preprocessing and normalization performed in R. Design and comparison matrices were employed for analyzing expression differences, and eBayes was used to smooth the results. The topTable function extracted significance statistics, while clusterProfiler was utilized for Gene Ontology and KEGG enrichment analysis [64], while clusterProfiler was utilized for Gene Ontology and KEGG enrichment analysis, the results were visualized via dot plots or bar plots. Analyses were performed in R (v4.2.1).

### Statistical analysis

Quantitative data were processed using the GraphPad Prism software (version 8.0.2). Following verification of normal distribution (Shapiro–Wilk test) and homogeneity of variance (Brown–Forsythe test), results were expressed as arithmetic mean ± standard error of the mean. Two-tailed *t* tests (with Welch’s correction) compared 2 groups. One-way analysis of variance (ANOVA) was first performed. For multiple group comparisons, one-way ANOVA was followed by Dunnett’s post hoc test when each treatment group was compared with a control group or by Tukey’s honestly significant difference test when all possible pairs of groups were compared. The significance levels are as follows: *P < 0.05, **P < 0.01, ***P < 0.001, and ****P < 0.0001.

## Ethical Approval

The study was approved by Xiangya Hospital, Central South University. The authors consented to publish the manuscript.

## Data Availability

The data and the code that support the findings of this study are available on reasonable request from the corresponding authors. The transcriptome data (RNA-seq data of HOXC10-depleted HDFs) are openly available in the GSA database (accession no. PRJCA045312); proteome data of mouse skin tissues are publicly available in the ProteomeXchange-iProX database (accession no. PXD067913).
