# Supplementary material for: HOXC10 Protects from Skin Aging by Targeting the FZD6/Wnt/β-Catenin Signaling Pathway
Source: Research (Wash D C). 2025 Nov 19;8:0976. doi: 10.34133/research.0976 (PMC12627330; doi:10.34133/research.0976)
Supplement: Supplementary 1 — Figs. S1 to S10 Tables S1 and S2 Supplementary Material 2 [file research.0976.f1.zip › Revised supplementary material 1.docx]

**HOXC10 protects from aging by targeting FZD6/Wnt/β-catenin signaling pathway**

**Yun Zhong^1,2,5#^, Yi Guo^1,2#^, Rui Mao^1,2^, Lei Zhou^2,3^, Fan Wang^1,2^, Xin Meng****^1,2^, Xiao Xin^1,2^ ,Haonan Yuan^1,2^, Yifan Zhang^1,2^, Zhili Deng^1,2,4^ , Wei Shi^1,2,4^ , Qian Wang^6^, Hongfu Xie^1,2,7^, Yiya Zhang^1,2,4 *^, Ji Li^1,2,4 *^**

1. Department of Dermatology, Xiangya Hospital, Central South University, Changsha, P.R. China.

2. Hunan key laboratory of aging biology, Xiangya Hospital, Central South University, Changsha, P.R. China.

3. Department of Dermatology, the Third Affiliated Hospital, Sun Yat-sen University, Guangzhou, P.R. China.

4. National Clinical Research Center for Geriatric Disorders, Xiangya Hospital, Central South University, Changsha, Hunan, P.R. China, 410008.

5. Department of Dermatology, Nanfang Hospital, Southern Medical University, Guangzhou, China.

6. Hunan Binsis Biotechnology Co.,Ltd

7. The First Hospital of Changsha, Changsha, 410005, China; The Affiliated Changsha Hospital of Xiangya School of Medicine, Central South University, Changsha, 410008, China.

*** Corresponding author.**

Ji Li: liji_xy@csu.edu.cn, Tel: +86 731 84327472;

Yiya Zhang: yiya0108@csu.edu.cn

## Supplementary figure


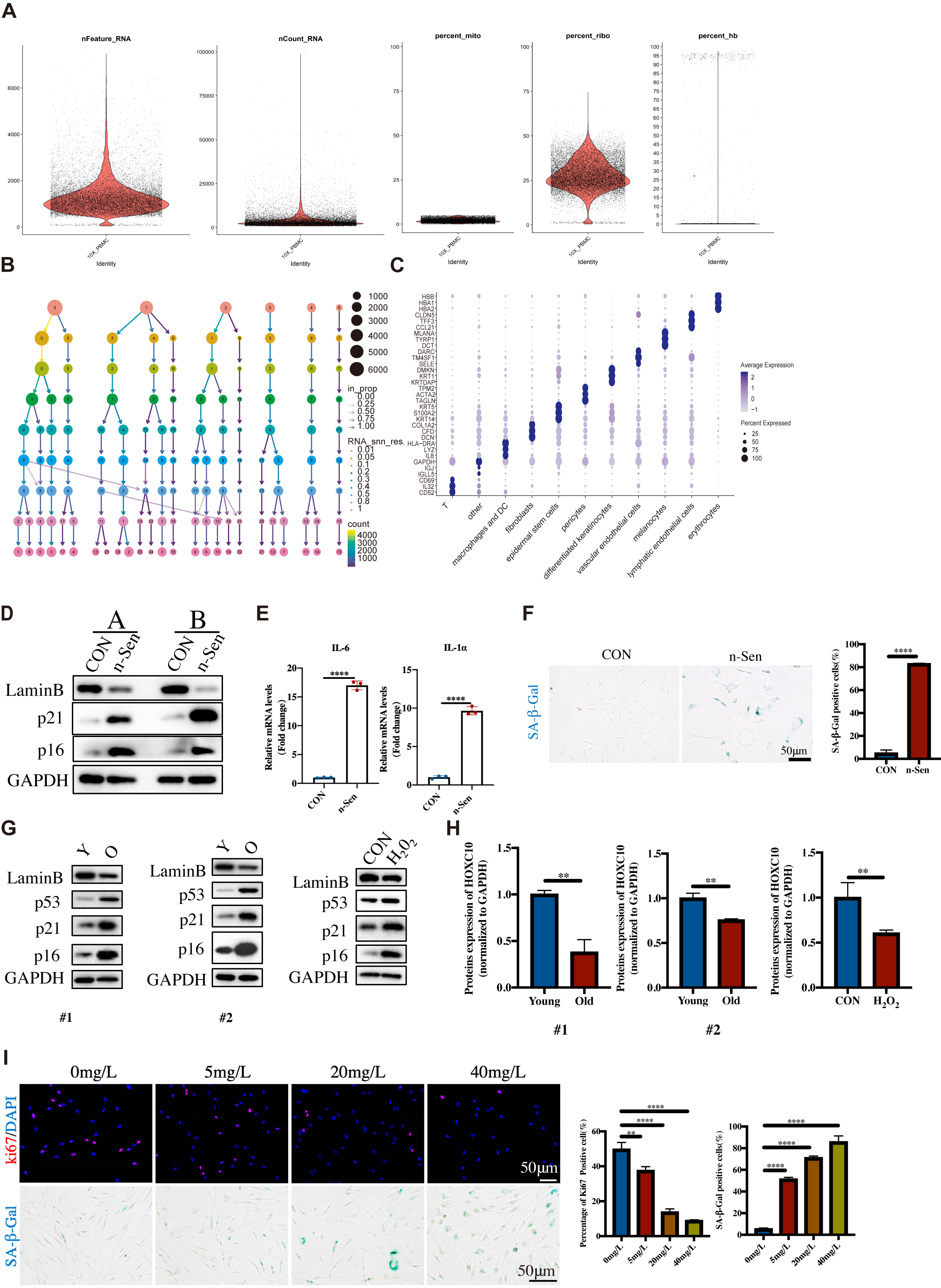


## Fig. S1 HOXC10 plays an important role in senescent cells and aging skin tissue.

(A) Ratiometric Rod-like Graph Displaying Mitochondria, Ribosomes, and Red Blood Cells Identified During Quality Control: Each dot represents a single cell. (B) Dendrogram from Cluster Tree Analysis: Varied colors signify different resolution levels, with resolution progressively increasing from top to bottom. (C) Dotplot of the Top 3 Highly Expressed Genes in Each Cell Type: Darker circles indicate higher gene expression, while larger circles denote a greater expression ratio of the gene in that particular cell type. (D-F) Verify that the n-Sen model is successfully constructed: protein levels of aging markers (D), mRNA levels of SASP-related genes (E), and SA-β-Gal staining (F). (G) The protein levels of aging-related markers verified that the passage senescence and H_2_O_2_-induced senescence models were successfully constructed. (H) Protein expression of HOXC10 in passage senescence and H_2_O_2_-Induced Senescence. (I) Immunofluorescence staining of ki67 and SA-β-Gal staining verified that the D-gal-induced senescence model was successfully constructed. Data are shown as mean ± SEM. *P < 0.05; **P < 0.01; ***P < 0.001.


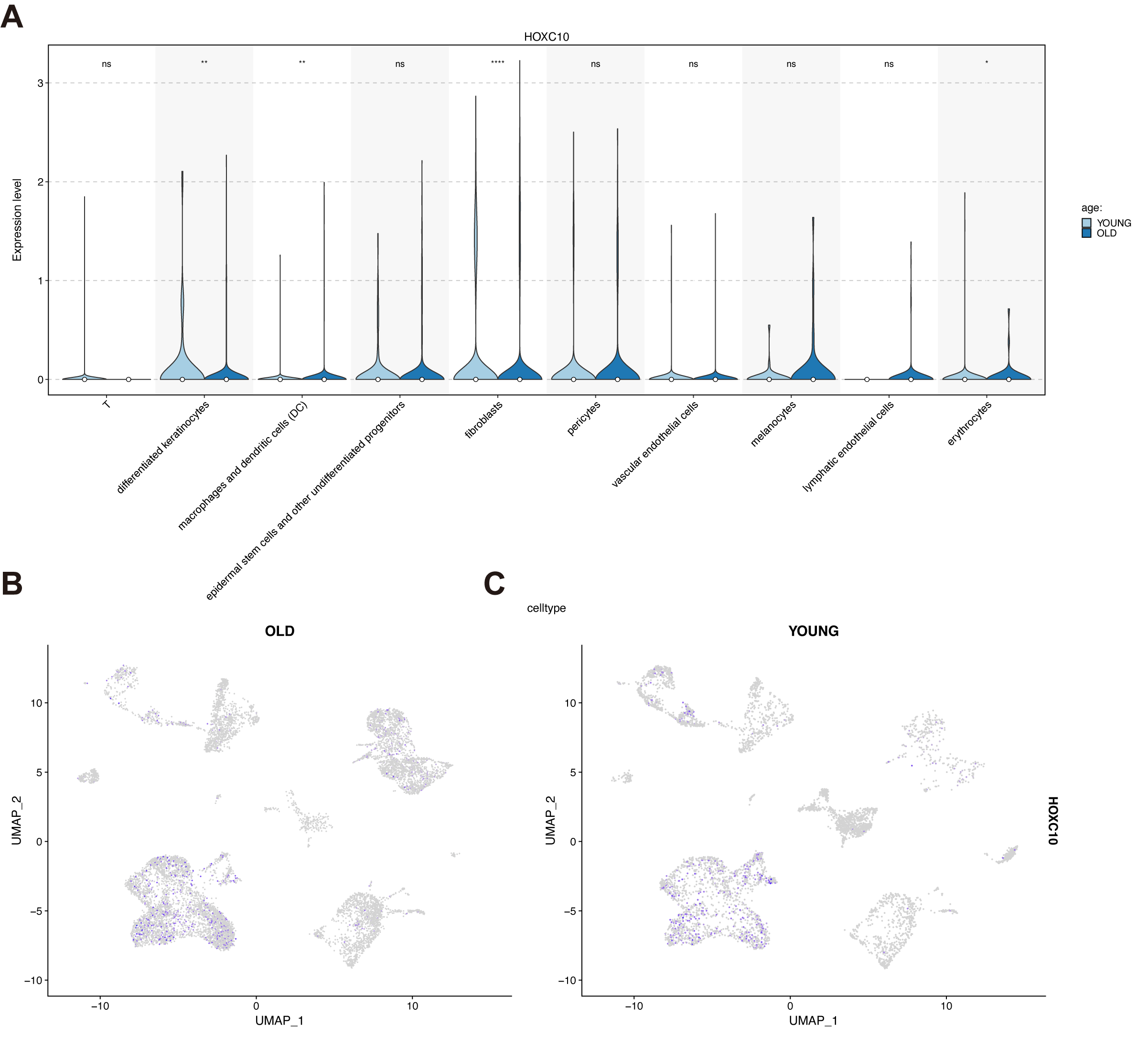


## Fig. S2 Differential Expression of HOXC10 Between Young and Aged Groups.

(A) Differential expression of HOXC10 between young and aged groups across various cell types; (B) FeaturePlot showing HOXC10 expression in the aged group; (C) FeaturePlot showing HOXC10 expression in the young group.


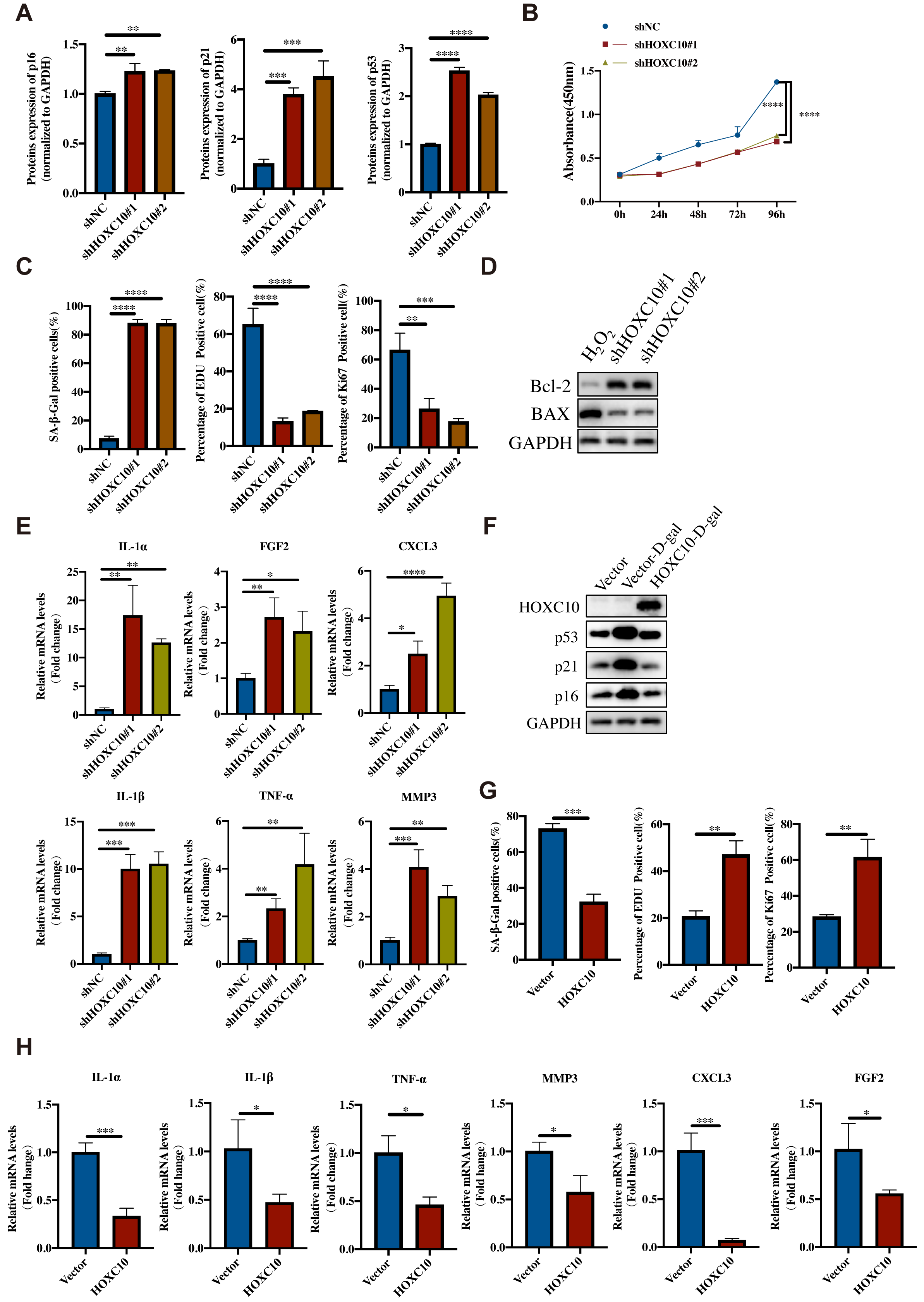


**Fig. S3 HOXC10 delayed HDFs senescence.**

(A) Protein expression of p53, p21 and p16 in shNC and shHOXC10 HDFs. (B)Cell proliferation after knockdown of HOXC10 measured by CCK8. (C) Quantification of ki67、Edu and SA-β-Gal positive cells in shNC and shHOXC10 HDF. (D) The proteins levels of BCL-2 and Bax after knockdown of HOXC10 by western blotting. (E) The mRNA expression levels of SASP after knockdown of HOXC10 by RT-qPCR. (F) HOXC10 was overexpressed while inducing the D-gal model, and the protein levels of HOXC10, p16, p21 and p53 were detected by western blotting. (G) Quantification of ki67、Edu and SA-β-Gal positive cells in Vector and HOXC10 HDF. (H) The mRNA expression levels of SASP after overexpression of HOXC10 by RT-qPCR. n=6 mice per group, mean age 2 month. Data are shown as mean ± SEM. *P < 0.05; **P < 0.01; ***P < 0.001.


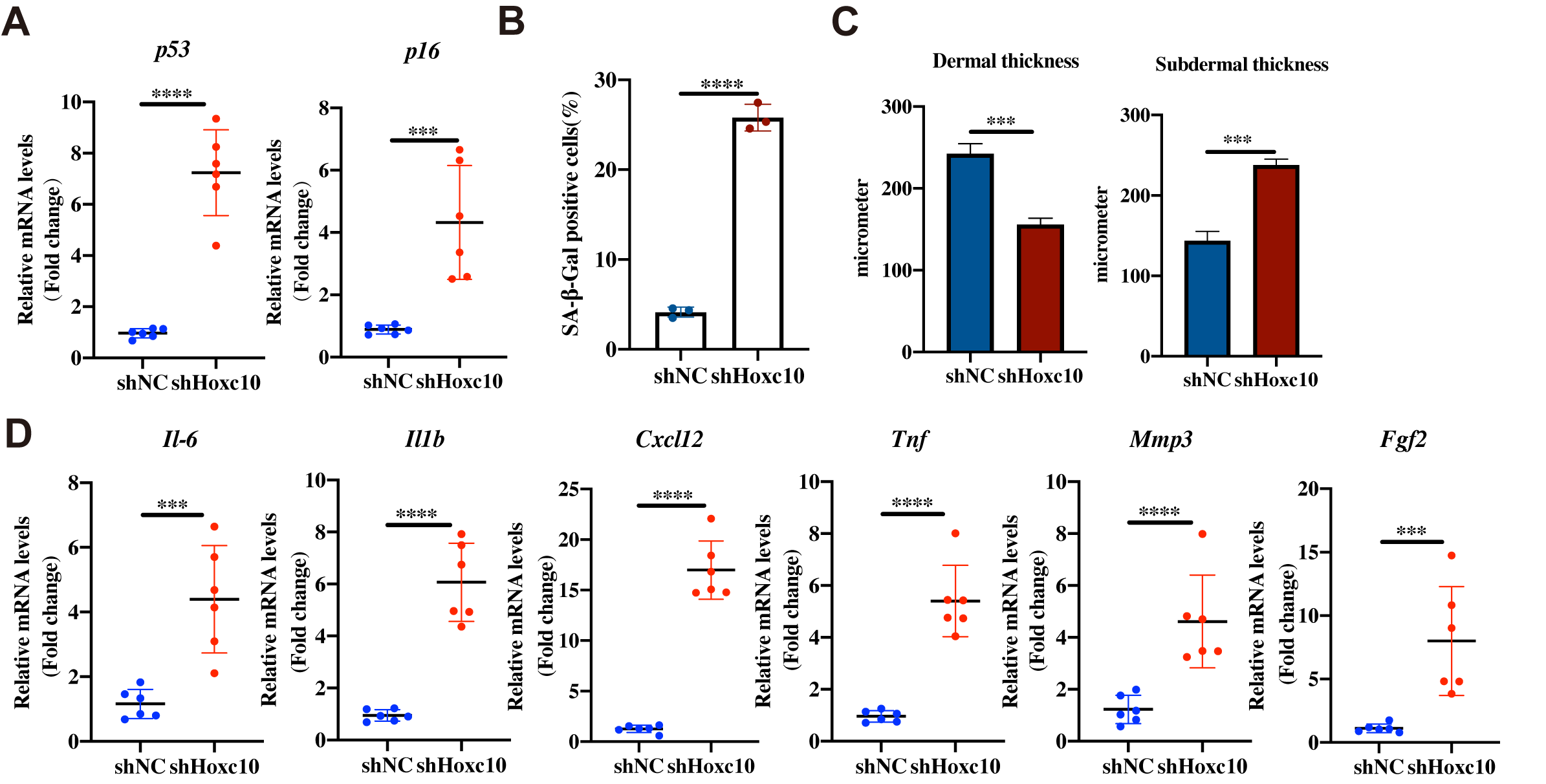


**Fig. S4 HOXC10 delayed skin aging.**

(A) The mRNA expression of p53, p16 in shHOXC10 skin of mice. (B) Statistics of β-Gal immunoflurescence staining in skin tissues. (C) Statistics of H&E staining in skin tissues. (D) The mRNA expression levels of SASP after knockdown of HOXC10 in skin by RT-qPCR. n=6 mice per group, mean age 2 month. Data are shown as mean ± SEM. *P < 0.05; **P < 0.01; ***P < 0.001.


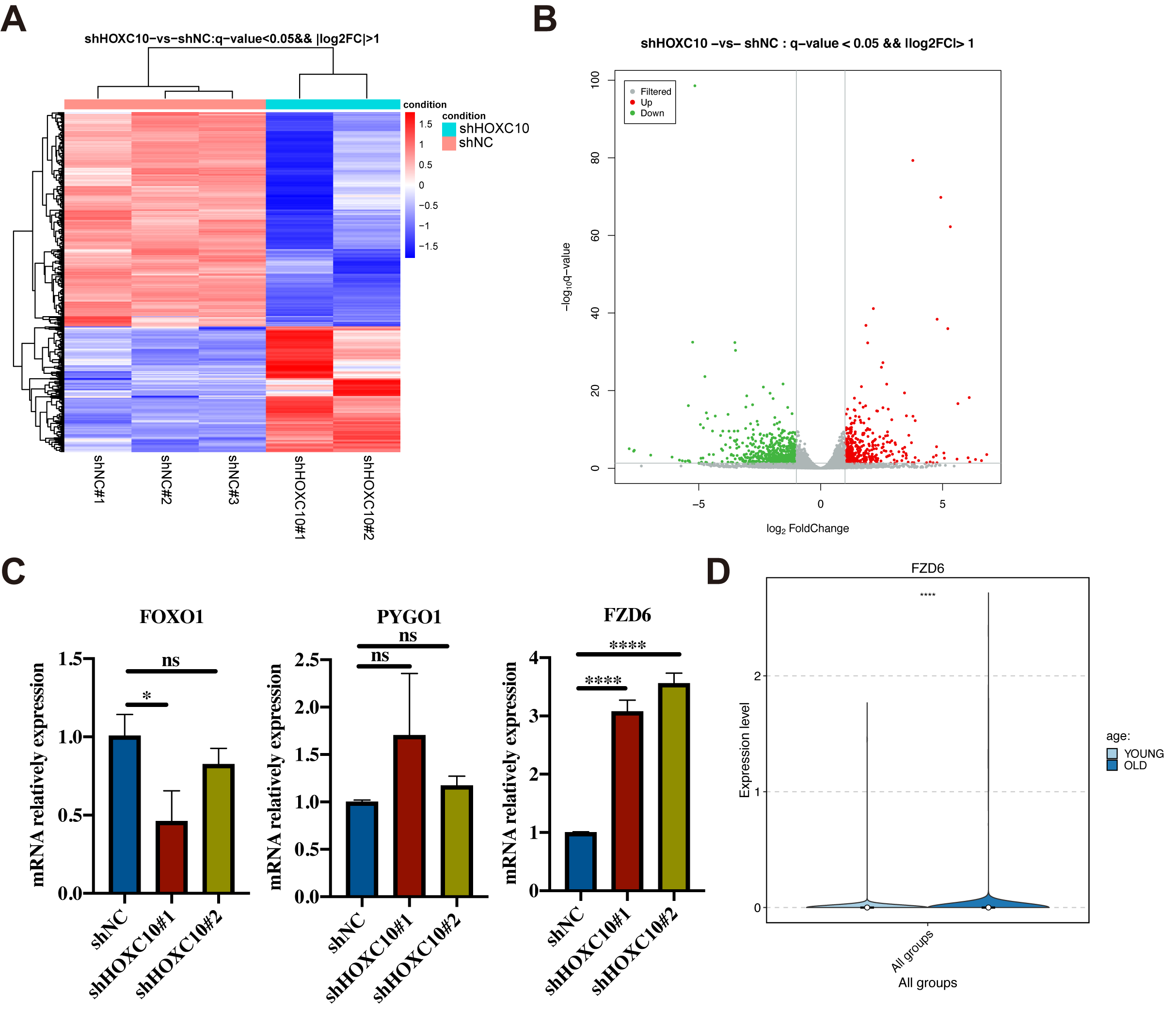


**Fig. S5 RNA-seq and experimental verification revealed FZD6 as a downstream gene regulated by HOXC10.**

(A, B) The volcano map and heatmap of DEGs. High or low expression is indicated by red or green in the digram. (C) Expression of FOXO1, PYGO1, FZD6 was examined by RT-qPCR following the knockdown of HOXC10. (D) Differential expression of FZD6 in young and aged samples. Data are shown as mean ± SEM. *P < 0.05; **P < 0.01; ***P < 0.001.


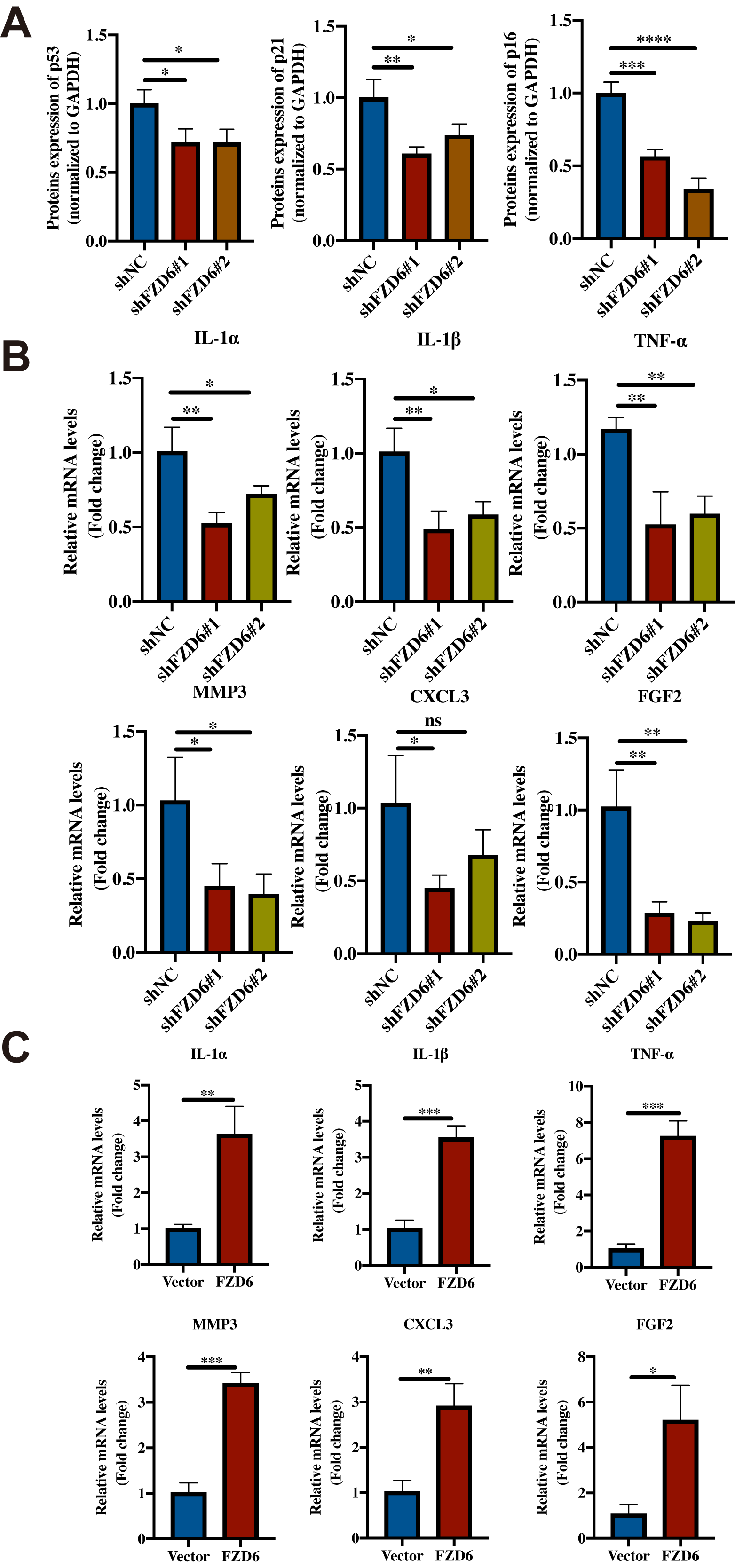


**Fig. S6 FZD6 Regulates SASP Expression in HDFs**

(A) The expression of SASP was measured by qRT-PCR in HDF treated with shNC and shFZD6. (B) The expression of SASP was measured by qRT-PCR in HDF treated with Vector and FZD6. Data are shown as mean ± SEM. *P < 0.05; **P < 0.01; ***P < 0.001.


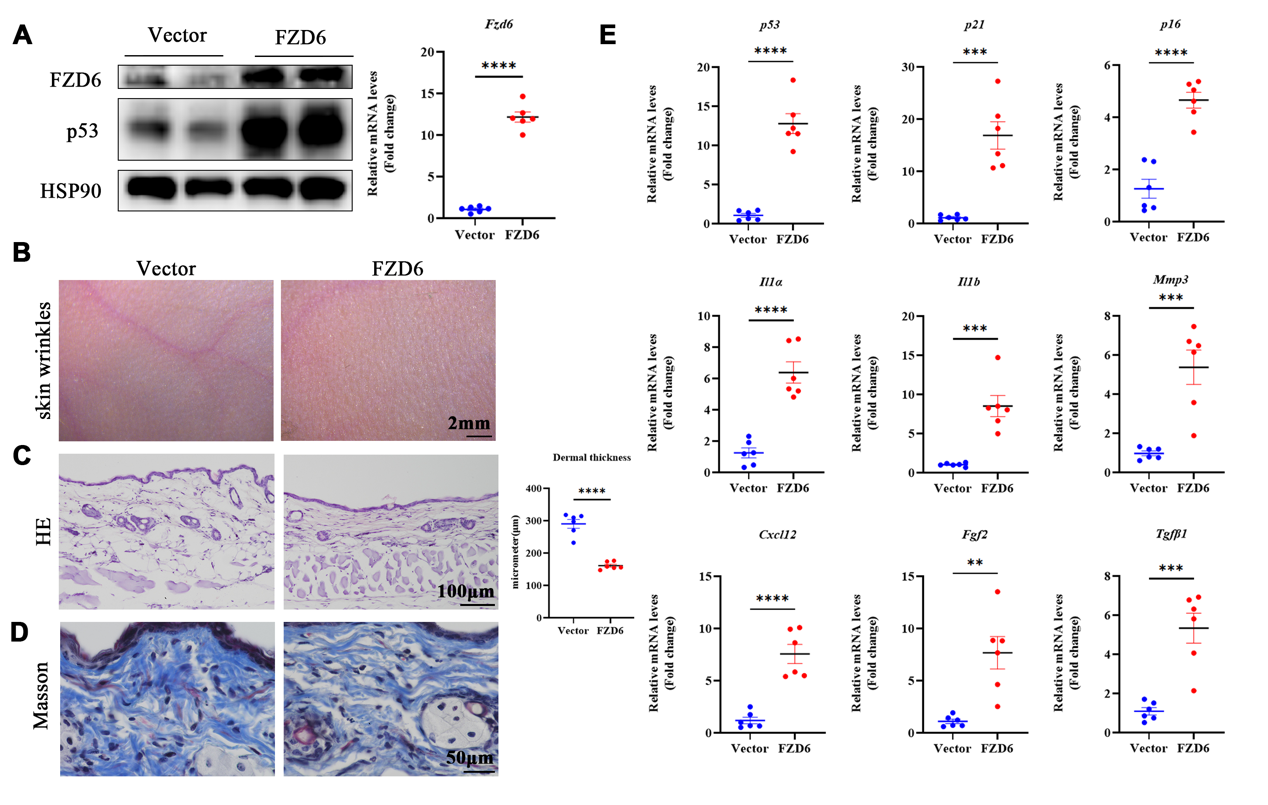


**Fig. S7 Overexpression of FZD6 delays skin aging.**

(A) The protein expression of FZD6 and p53(left), and the mRNA expression of FZD6 in skin of mice. (n=6 mice per group, mean age 2 month). (B) Representative images of skin texture from different groups. scale bars: 2 mm. (C) Representative images (left) and statistics (right) of H&E staining from different groups. scale bars: 100 μm. (D) Representative images of Masson staining from different groups. scale bars: 50 μm. (E) The expression of *p53*, *p21*, *p16*, *Il1α*, *Il1β*, *Mmp3*, *Cxcl12*, *Fgf2,* and *Tnfα* was verified at the mRNA level. Data are shown as mean ± SEM. *P < 0.05; **P < 0.01; ***P < 0.001.


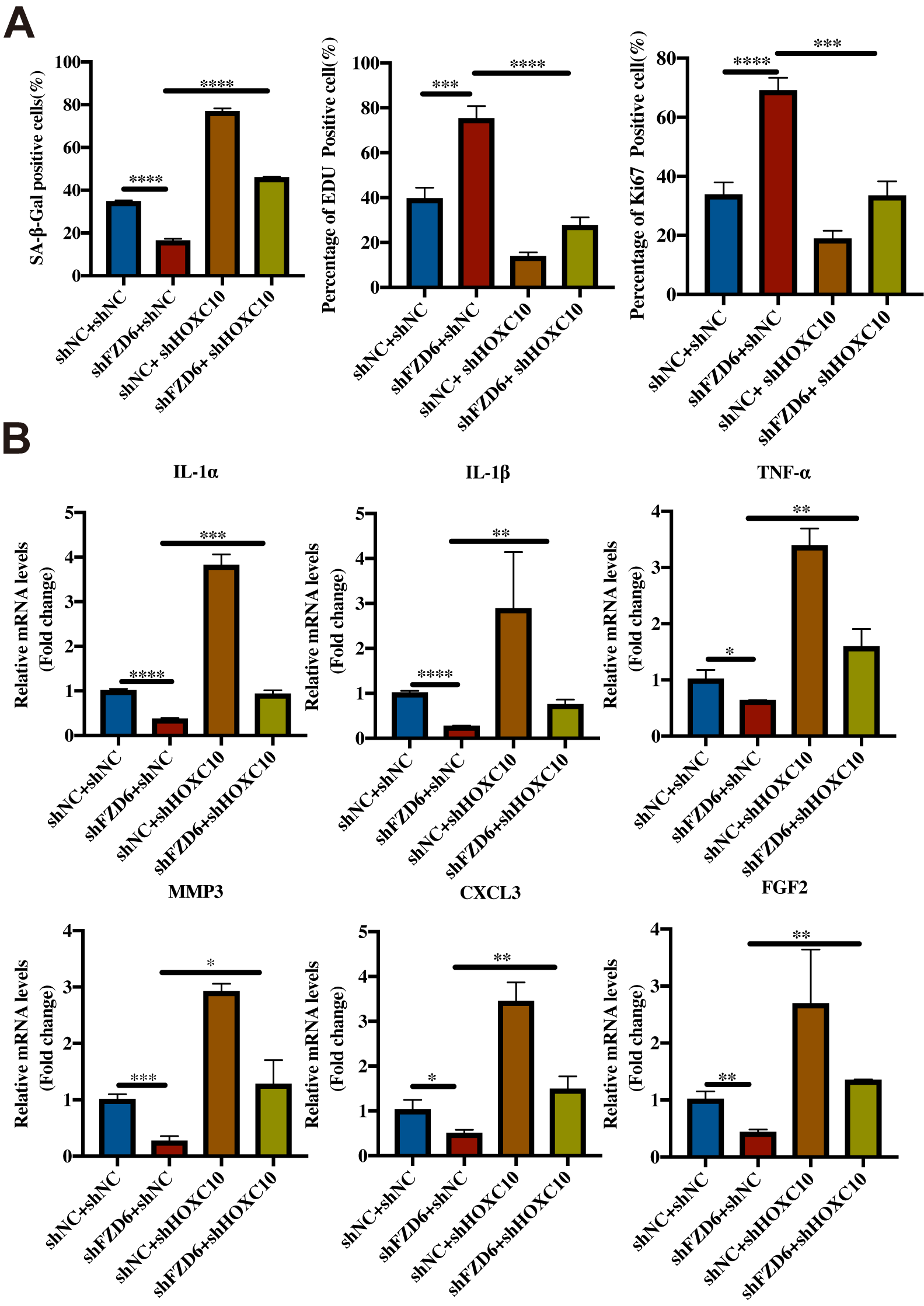


**Fig. S8 FZD6 was involved in HOXC10-mediated cellular senescence in HDF.**

(A) Quantification of ki67, Edu and SA-β-Gal positive cells in shNC+ shNC, shFZD6+shNC, shNC+shHOXC10 and shFZD6+shHOXC10 treated HDF. (B) The expression of SASP was measured by qRT-PCR to assess the reversed effect of FZD6 in HOXC10-mediated senescence. Data are shown as mean ± SEM. *P < 0.05; **P < 0.01; ***P < 0.001.


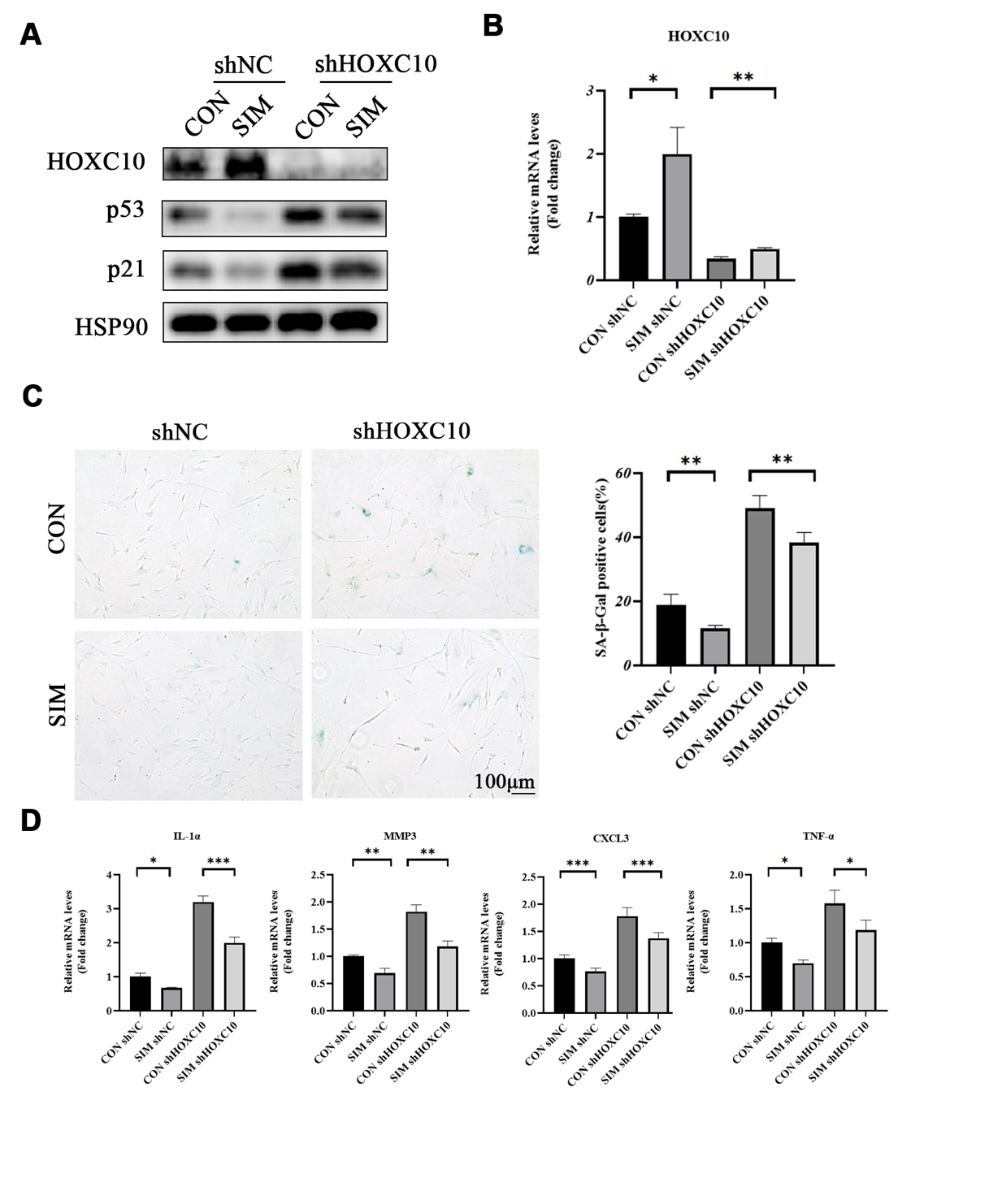


**Fig. S9 Simvastatin can partially delay the HDFs senescence induced by the knockdown of HOXC10.**

Young-passages HDFs (PD<15) were cultured in medium containing SIM (100 nM) or normal medium and then infected with shHOXC10 or negative control shNC after 5-6 passages. (A) The proteins levels of HOXC10, p53 and p21 by western blotting. (B) The mRNA expression levels of HOXC10 by RT-qPCR. (C) Representative images (left) and statistics (righ) of SA-β-Gal staining. scale bars: 100 μm. (D)The mRNA expression levels of IL-1α, MMP3, CXCL3, and TNF-α by qRT-PCR. Data are shown as mean ± SEM. *P < 0.05; **P < 0.01; ***P < 0.001.


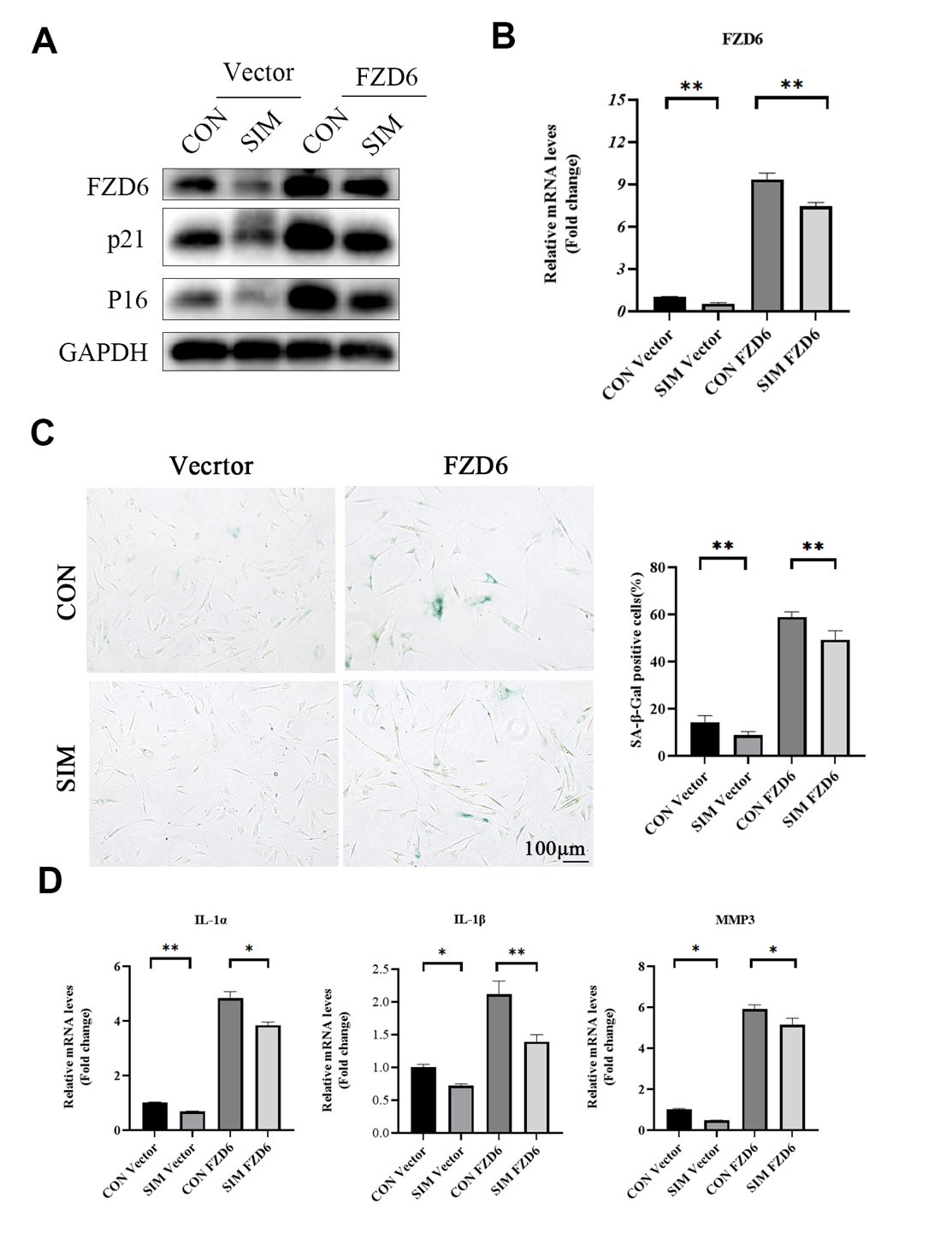


**Fig. S10 Simvastatin can partially delay the HDFs senescence induced by the Overexpression of FZD6.**

Young-passages HDFs (PD<15) were cultured in medium containing SIM (100 nM) or normal medium and then infected with FZD6 or Verctor after 5-6 passages. (A) The proteins levels of FZD6, p21 and p16 by western blotting. (B) The mRNA expression levels of FZD6 by qRT-PCR. (C) Representative images (left) and statistics (righ) of SA-β-Gal staining. scale bars: 100 μm. (D) The mRNA expression levels of IL-1α, IL-1β, and MMP3 by qRT-PCR. Data are shown as mean ± SEM. *P < 0.05; **P < 0.01; ***P < 0.001.

## Appendix Table 1. List of primer sequences in this study.

| Gene symbol | Forward primer | Reverse primer |
| --- | --- | --- |
| **Primers for RT-qPCR:** | | |
| Human-GAPDH | GGAGCGAGATCCCTCCAAAAT | GGCTGTTGTCATACTTCTCATGG |
| Human-HOXC10 | ACATGCCCTCGCAATGTAACT | GAGAGGTAGGACGGATAGGTG |
| Human-FZD6 | ATGGCCTACAACATGACGTTT | GTTTACGACAAGGTGGAACCA |
| Human-β-CATENIN | ATGGAGCCGGACAGAAAAGC | AGGAAGGGACTCACCGTTC |
| Human-AXIN2 | GGGGGAGACAGGAAGTTTTA | AACATGGTCAACCCTCAAGA |
| Human- GSK-3β | ACAACAGTGGTGGCAACTCC | TTCTTGATGGCGACCAGTTCT |
| Human-FOXO1 | TCGTCATAATCTGTCCCTACACA | CGGCTTCGGCTCTTAGCAAA |
| Human-PYGO1 | CAGCTCCCGCTTACAAAGTTT | GGTCCCTGTGTATTTGCCTTG |
| Human-IL-6 | CCTGAACCTTCCAAAGATGGC | TTCACCAGGCAAGTCTCCTCA |
| Human-IL-1β | ATGATGGCTTATTACAGTGGCAA | GTCGGAGATTCGTAGCTGGA |
| Human-IL-1α | ACTCACCTCTTCAGAACGAATTG | CCATCTTTGGAAGGTTCAGGTTG |
| Human-IL-8 | TTTTGCCAAGGAGTGCTAAAGA | AACCCTCTGCACCCAGTTTTC |
| Human-CXCL3 | CGCCCAAACCGAAGTCATAG | GCTCCCCTTGTTCAGTATCTTTT |
| Human-FGF2 | AGAAGAGCGACCCTCACATCA | CGGTTAGCACACACTCCTTTG |
| Human-MMP3 | AGTCTTCCAATCCTACTGTTGCT | TCCCCGTCACCTCCAATCC |
| Human-TNF-α | CCTCTCTCTAATCAGCCCTCTG | GAGGACCTGGGAGTAGATGAG |
| Mouse-Gapdh | AGGTCGGTGTGAACGGATTTG | TGTAGACCATGTAGTTGAGGTCA |
| Mouse-Hoxc10 | ATGACATGCCCTCGCAATGTA | CCCCGCAGTTGAAGTCACTC |
| Mouse-Fzd6 | TCTGCCCCTCGTAAGAGGAC | GGGAAGAACGTCATGTTGTAAGT |
| Mouse-Il1β | GCAACTGTTCCTGAACTCAACT | ATCTTTTGGGGTCCGTCAACT |
| Mouse-Cxcl2 | CCAACCACCAGGCTACAGG | GCGTCACACTCAAGCTCTG |
| Mouse-  Il6 | TTCTCCTGGCAAAGACGGACTCAA | AGGAAGCTGAAGTCATAACCGCCA |
| Mouse-Fgf2 | GCGACCCACACGTCAAACTA | TCCCTTGATAGACACAACTCCTC |
| Mouse-Mmp3 | GGCCTGGAACAGTCTTGGC | TGTCCATCGTTCATCATCGTCA |
| Mouse-Tnfa | CAGGCGGTGCCTATGTCTC | CGATCACCCCGAAGTTCAGTAG |
| Mouse-p16 | CGCAGGTTCTTGGTCACTGT | TGTTCACGAAAGCCAGAGCG |
| Mouse-p53 | ATGAACCGCCGACCTATCC | GGCAGGCACAAACACGAAC |
| **Primer for promoter:**   \| FZD6  promoter \| TAAGCAGAGCTCAAAATCTCTTCCAGAAGATAGAAGCAGGGG \| TGCTTACTCGAGTATCCGGGTCCGGAGGGGTT \| \| --- \| --- \| --- \| \| P1 promoter \| CGAGCTCGATCTCTTCCAGAAGATAG \| CCGCTCGAGCGGTAGAAGGATTTTTTGAAGC \| \| P2 promoter \| CGAGCTCGCTATAGAGGAACAAAAATAAAGAAT \| CCGCTCGAGCGGTAGAAGGATTTTTTGAAGCTC \| \| P3 promoter \| CGAGCTCGCATTCCTTTTCTGCAACAG \| CCGCTCGAGCGGTAGAAGGATTTTTTGAAGCTC \|   **Primer for Chip-qPCR:**   \| Site1+2 \| ACAGCCAGTGCTCAATTAACG \| AGGGGCATAGTTTCCTCACA \| \| --- \| --- \| --- \| \| Site3 \| TTGTGAGGAAACTATGCCCCTT \| TTTGGGGTACCAACTCTGTCA \| \| Site4 \| AGCAGTTCAACTTCCTATTAGGGT \| GGTCACCAACTGGATGCTCA \| \| Site5 \| TGACCAGGCTCCCCGAATTA \| GCTTTGTAAAGCCAATTGCCCA \|   **Primer for shRNA:** | | |
| shNC | CCGGCAACAAGATGAAGAGCACCAACTCGAGTTGGTGCTCTTCATCTTGTTGTTTTTG | AATTCAAAAACAACAAGATGAAGAGCACCAACTCGAGTTGGTGCTCTTCATCTTGTTG |
| shHOXC10#1 | CCGGCTGGAGATTAGCAAGACCATTCTCGAGAATGGTCTTGCTAATCTCCAGTTTTTG | AATTCAAAAACTGGAGATTAGCAAGACCATTCTCGAGAATGGTCTTGCTAATCTCCAG |
| shHOXC10#2 | CCGGACCTAGTGTCAAGGAGGAGAACTCGAGTTCTCCTCCTTGACACTAGGTTTTTTG | AATTCAAAAAACCTAGTGTCAAGGAGGAGAACTCGAGTTCTCCTCCTTGACACTAGGT |
| shHoxc10 | CCGGCTTACAGACAGACAAGTCAAACTCGAGTTTGACTTGTCTGTCTGTAAGTTTTTG | AATTCAAAAACTTACAGACAGACAAGTCAAACTCGAGTTTGACTTGTCTGTCTGTAAG |
| shFZD6#1 | CCGGCCCATGTCCTTATCAGGCAAACTCGAGTTTGCCTGATAAGGACATGGGTTTTTG | AATTCAAAAACCCATGTCCTTATCAGGCAAACTCGAGTTTGCCTGATAAGGACATGGG |
| shFZD6#2 | CCGGCCCTAATCTGATGGGTCATTACTCGAGTAATGACCCATCAGATTAGGGTTTTTG | AATTCAAAAACCCTAATCTGATGGGTCATTACTCGAGTAATGACCCATCAGATTAGGG |
| LV-HOXC10 | GGCGGTGGTGGATCCGAATTCTGGAAGGGCTAATTCACTCCC | GGTACTAGTTCTAGACTCGAGACTACTAAGTTTGTAGTACATATTTAACAAATACAA |
| LV- FZD6 | CGGAATTCATGGAAATGTTTACATTTTT | TGCTTAGCGGCCGCTCAAGTATCTGAATGACA |

## Appendix Table 2. JASPAR predicts possible binding sites between FZD6 promoter and HOXC10.

| Matrix ID | Name | Score | Relative score | Sequence ID | Start | End | Strand | Predicted sequence |
| --- | --- | --- | --- | --- | --- | --- | --- | --- |
| [MA0905.1](https://jaspar.elixir.no/matrix/MA0905.1) | MA0905.1.HOXC10 | 13.2188 | 0.97434 | NC_000008.11:103296494-103298593 | 341 | 350 | + | ATCATAAAAA |
| [MA0905.1](https://jaspar.elixir.no/matrix/MA0905.1) | MA0905.1.HOXC10 | 9.25466 | 0.90736 | NC_000008.11:103296494-103298593 | 1178 | 1187 | + | CTCATTAAGT |
| [MA0905.1](https://jaspar.elixir.no/matrix/MA0905.1) | MA0905.1.HOXC10 | 9.0387 | 0.90371 | NC_000008.11:103296494-103298593 | 939 | 948 | + | ATAATTAAAT |
| [MA0905.1](https://jaspar.elixir.no/matrix/MA0905.1) | MA0905.1.HOXC10 | 7.04886 | 0.8701 | NC_000008.11:103296494-103298593 | 1667 | 1676 | + | ATCATTACAA |
| [MA0905.1](https://jaspar.elixir.no/matrix/MA0905.1) | MA0905.1.HOXC10 | 5.95076 | 0.85154 | NC_000008.11:103296494-103298593 | 1810 | 1819 | + | ATCAATAAAA |
| [MA0905.1](https://jaspar.elixir.no/matrix/MA0905.1) | MA0905.1.HOXC10 | 5.55884 | 0.84492 | NC_000008.11:103296494-103298593 | 1165 | 1174 | + | ATCACAAAAA |
| [MA0905.1](https://jaspar.elixir.no/matrix/MA0905.1) | MA0905.1.HOXC10 | 4.06638 | 0.81971 | NC_000008.11:103296494-103298593 | 123 | 132 | + | GATGTAAAAA |
| [MA0905.1](https://jaspar.elixir.no/matrix/MA0905.1) | MA0905.1.HOXC10 | 3.64859 | 0.81265 | NC_000008.11:103296494-103298593 | 1220 | 1229 | + | GTAGAAAATT |
| [MA0905.1](https://jaspar.elixir.no/matrix/MA0905.1) | MA0905.1.HOXC10 | 3.59476 | 0.81174 | NC_000008.11:103296494-103298593 | 577 | 586 | + | GCCTTTAAAA |
| [MA0905.1](https://jaspar.elixir.no/matrix/MA0905.1) | MA0905.1.HOXC10 | 3.22607 | 0.80551 | NC_000008.11:103296494-103298593 | 1889 | 1898 | + | TTAATAAAAG |
| [MA0905.1](https://jaspar.elixir.no/matrix/MA0905.1) | MA0905.1.HOXC10 | 3.15924 | 0.80438 | NC_000008.11:103296494-103298593 | 1657 | 1666 | + | AACGTTAACC |
| [MA0905.1](https://jaspar.elixir.no/matrix/MA0905.1) | MA0905.1.HOXC10 | 3.13749 | 0.80401 | NC_000008.11:103296494-103298593 | 956 | 965 | + | AAAATAAAAC |
| [MA0905.1](https://jaspar.elixir.no/matrix/MA0905.1) | MA0905.1.HOXC10 | 3.1141 | 0.80362 | NC_000008.11:103296494-103298593 | 1726 | 1735 | + | GTAATTTATC |
| [MA0905.1](https://jaspar.elixir.no/matrix/MA0905.1) | MA0905.1.HOXC10 | 3.10124 | 0.8034 | NC_000008.11:103296494-103298593 | 1110 | 1119 | + | GTTTTTAAAT |
| [MA0905.1](https://jaspar.elixir.no/matrix/MA0905.1) | MA0905.1.HOXC10 | 2.9012 | 0.80002 | NC_000008.11:103296494-103298593 | 1095 | 1104 | + | ATTTTAAAAA |

| **Matrix ID** | **Name** | **Score** | **Relative score** | **Sequence ID** | **Start** | **End** | **Strand** | **Predicted sequence** |
| --- | --- | --- | --- | --- | --- | --- | --- | --- |
| [MA0905.1](https://jaspar.elixir.no/matrix/MA0905.1) | MA0905.1.HOXC10 | 13.2188 | 0.97434 | NC_000008.11:103296494-103298593 | 341 | 350 | + | ATCATAAAAA |
| [MA0905.1](https://jaspar.elixir.no/matrix/MA0905.1) | MA0905.1.HOXC10 | 9.25466 | 0.90736 | NC_000008.11:103296494-103298593 | 1178 | 1187 | + | CTCATTAAGT |
| [MA0905.1](https://jaspar.elixir.no/matrix/MA0905.1) | MA0905.1.HOXC10 | 9.0387 | 0.90371 | NC_000008.11:103296494-103298593 | 939 | 948 | + | ATAATTAAAT |
| [MA0905.1](https://jaspar.elixir.no/matrix/MA0905.1) | MA0905.1.HOXC10 | 7.04886 | 0.8701 | NC_000008.11:103296494-103298593 | 1667 | 1676 | + | ATCATTACAA |
| [MA0905.1](https://jaspar.elixir.no/matrix/MA0905.1) | MA0905.1.HOXC10 | 5.95076 | 0.85154 | NC_000008.11:103296494-103298593 | 1810 | 1819 | + | ATCAATAAAA |
| [MA0905.1](https://jaspar.elixir.no/matrix/MA0905.1) | MA0905.1.HOXC10 | 5.55884 | 0.84492 | NC_000008.11:103296494-103298593 | 1165 | 1174 | + | ATCACAAAAA |
| [MA0905.1](https://jaspar.elixir.no/matrix/MA0905.1) | MA0905.1.HOXC10 | 4.06638 | 0.81971 | NC_000008.11:103296494-103298593 | 123 | 132 | + | GATGTAAAAA |
| [MA0905.1](https://jaspar.elixir.no/matrix/MA0905.1) | MA0905.1.HOXC10 | 3.64859 | 0.81265 | NC_000008.11:103296494-103298593 | 1220 | 1229 | + | GTAGAAAATT |
| [MA0905.1](https://jaspar.elixir.no/matrix/MA0905.1) | MA0905.1.HOXC10 | 3.59476 | 0.81174 | NC_000008.11:103296494-103298593 | 577 | 586 | + | GCCTTTAAAA |
| [MA0905.1](https://jaspar.elixir.no/matrix/MA0905.1) | MA0905.1.HOXC10 | 3.22607 | 0.80551 | NC_000008.11:103296494-103298593 | 1889 | 1898 | + | TTAATAAAAG |
| [MA0905.1](https://jaspar.elixir.no/matrix/MA0905.1) | MA0905.1.HOXC10 | 3.15924 | 0.80438 | NC_000008.11:103296494-103298593 | 1657 | 1666 | + | AACGTTAACC |
| [MA0905.1](https://jaspar.elixir.no/matrix/MA0905.1) | MA0905.1.HOXC10 | 3.13749 | 0.80401 | NC_000008.11:103296494-103298593 | 956 | 965 | + | AAAATAAAAC |
| [MA0905.1](https://jaspar.elixir.no/matrix/MA0905.1) | MA0905.1.HOXC10 | 3.1141 | 0.80362 | NC_000008.11:103296494-103298593 | 1726 | 1735 | + | GTAATTTATC |
| [MA0905.1](https://jaspar.elixir.no/matrix/MA0905.1) | MA0905.1.HOXC10 | 3.10124 | 0.8034 | NC_000008.11:103296494-103298593 | 1110 | 1119 | + | GTTTTTAAAT |
| [MA0905.1](https://jaspar.elixir.no/matrix/MA0905.1) | MA0905.1.HOXC10 | 2.9012 | 0.80002 | NC_000008.11:103296494-103298593 | 1095 | 1104 | + | ATTTTAAAAA |
